# MAZ promotes prostate cancer bone metastasis through transcriptionally activating the KRas-dependent RalGEFs pathway

**DOI:** 10.1186/s13046-019-1374-x

**Published:** 2019-09-05

**Authors:** Qing Yang, Chuandong Lang, Zhengquan Wu, Yuhu Dai, Shaofu He, Wei Guo, Shuai Huang, Hong Du, Dong Ren, Xinsheng Peng

**Affiliations:** 1 Department of Orthopaedic Surgery, The First Affiliated Hospital, Sun Yat-sen University, 58# Zhongshan 2rd Road, Guangzhou, 510080 Guangdong Province China; 2 Guangdong Provincial Key Laboratory of Orthopedics and Traumatology, Guangzhou, 510080 Guangdong Province China; 3 Department of Radiology, The First Affiliated Hospital, Sun Yat-sen University, Guangzhou, 510080 Guangdong Province China; 4 Department of Orthopaedic Surgery, The Second Affiliated Hospital of Guangzhou Medical University, Guangzhou, 510260 China; 5 Department of Pathology, The First People’s Hospital of Guangzhou City, Guangzhou, 510180 Guangdong China

**Keywords:** MAZ, Bone metastasis, Prostate cancer, Ras signalling, And RalGEFs

## Abstract

**Background:**

Clinically, prostate cancer (PCa) exhibits a high avidity to metastasize to bone. Myc-associated zinc-finger protein (MAZ) is a well-documented oncogene involved in the progression and metastasis of multiple cancer types, even in PCa. However, the clinical significance and biological roles of MAZ in bone metastasis of PCa remain unclear.

**Methods:**

MAZ expression was examined in PCa tissues with bone metastasis, PCa tissues without bone metastasis and metastatic bone tissues by real-time PCR and immunohistochemistry (IHC), respectively. Statistical analysis was performed to evaluate the clinical correlation between MAZ expression and clinicopathological features and bone metastasis-free survival in PCa patients. Biological roles of MAZ in bone metastasis of PCa were investigated both in vitro by transwell assay, and in vivo by a mouse model of left cardiac ventricle inoculation. The bioinformatics analysis, western blot, pull-down assays, chromatin immunoprecipitation (ChIP) and luciferase reporter assays were applied to demonstrate and examine the relationship between MAZ and its potential downstream signalling pathway. TaqMan copy number assay was performed to identify the underlying mechanism responsible for MAZ overexpression in PCa tissues.

**Results:**

MAZ expression is elevated in PCa tissues with bone metastasis compared with that in PCa tissues without bone metastasis, and is further increased in metastatic bone tissues. High expression of MAZ positively correlates with poor overall and bone metastasis-free survival in PCa patients. Upregulating MAZ elevates, while silencing MAZ represses the invasion and migration abilities of PCa cells in vitro and bone metastasis ability in vivo. Our results further reveal that MAZ promotes bone metastasis of PCa dependent on KRas signalling, although MAZ transcriptionally upregulates KRas and HRas expression, where the Ral guanine nucleotide exchange factor (RalGEF) signaling is responsible for the different roles of KRas and HRas in mediating the pro-bone metastasis of MAZ in PCa. Finally, our results indicate that recurrent gains contribute to MAZ overexpression in a small portion of PCa tissues.

**Conclusion:**

These results indicate that the MAZ/Kras/ RalGEF signalling axis plays a crucial role in promoting PCa cell bone metastasis, suggesting a potential therapeutic utility of MAZ in bone metastasis of PCa.

**Electronic supplementary material:**

The online version of this article (10.1186/s13046-019-1374-x) contains supplementary material, which is available to authorized users.

## Novelty and impact

Prostate cancer (PCa) metastasis is the major determinant of cancer-related death and life quality of patients. A hallmark of PCa metastasis is the predominant propensity of bone metastasis. By in vitro and in vivo experiments, our research firstly proves that MAZ/Kras/ RalGEFs signaling axis plays an important role in the bone metastasis of PCa, suggesting a potential therapeutic utility of MAZ in bone metastasis of PCa.

## Background

Prostate cancer (PCa) metastasis is the major determinant of cancer-related death and quality of life of patients [1]. A distinguishing feature of PCa metastasis is the predominant susceptibility to bone metastasis [2]. Unlike liver, lung and brain tissues, bone marrow contains less arterial blood flow; thus, the hemodynamics determining metastatic colonization may not be important organ-specific factors contributing to bone metastasis [3, 4]. Therefore, unveiling the specific molecular mechanism underlying bone metastasis in PCa has become an urgent need.

To date, several cellular signalling pathways have been confirmed to be closely related to bone metastases of PCa based on a large amount of literature [5, 6], such as TGF-β [7], Wnt [8], NF-kB [9], EGFR [10], and Notch [11]. Notably, accumulating evidence has focused great attention on the crucial role of aberrant activation of RAS signaling in bone metastasis of PCa [12, 13]. Ras proteins are prototypical G-proteins that have been shown to play an important role in signal transduction, proliferation, and malignant transformation [14]. The Ras gene family consists of 3 functional genes, H-Ras, K-Ras and N-Ras, which primarily regulate multiple downstream signalling pathways, including the Ras–Raf–mitogen-activated and extracellular-signal regulated kinase kinase (MEK)–extracellular signal-regulated kinase (ERK) cascade, phosphatidylinositol 3-kinase (PI3K), and members of the Ral guanine nucleotide exchange factor (RalGEF) family [15]. All three of these signalling pathways have been proven to be critical for bone metastasis in various types of cancer, including PCa. For example, Ni et al. have informed that PI3K/Akt signaling -mediated stabilization of histone methyltransferase WHSC1 greatly raised bone metastasis in PCa [16]. In addition, Yin et al. demonstrated that activation of the RalGEF/Ral pathway promotes prostate cancer metastasis to bone [12]. Suppression of ERK signaling by XRP44X, an inhibitor of Ras/Erk signalling, effectively inhibits bone metastasis of PCa cells in an intracardiac injection mouse model [17]. Although mutations of activating Ras occur in ~ 30% of human cancers, Ras mutations in prostate cancer are infrequent [18, 19]. Interestingly, several lines of evidence have shown that transcriptional regulation is another primary mechanism accountable for the activation of Ras signalling in several types of cancer [20–22]. Therefore, it is of paramount importance to identify the underlying transcription factor responsible for constitutive activation of Ras signalling in bone metastasis of PCa.

The Myc-associated zinc-finger protein (MAZ) has been recognized as a transcription factor that binds to a GA box (GGGAGGG) at the ME1a1 site, to the attenuator region of P2 within the first exon of the c-myc gene and to a related sequence that participates in the termination of gene transcription of complement 2 (C2) [23, 24]. The MAZ gene is located on chromosome 16p11.2 and encodes a single, unique gene [24, 25]. Despite being ubiquitous in human tissue, the MAZ expression level varies depending on the organ. The mRNA expression levels of MAZ in the human heart, placenta, pancreas, thymus, prostate, testis, colon, peripheral blood leukocytes, thyroid, and adrenal gland are higher than those in other tissues such as bone marrow [25]. In cancers, MAZ is generally highly expressed in a variety of human tumors, which further promotes the development, progression, and metastasis of cancer by transcriptionally activating multiple downstream target genes [26–29]. In PCa, a marked increase in MAZ promotes proliferation and metastasis of PCa through reciprocal regulation of androgen receptor [26], implicating MAZ in the metastatic phenotype of PCa. However, the clinical significance and biological function of MAZ in bone metastasis of PCa remain to be elucidated.

In the present study, the expression levels of MAZ increased steadily from nonbone metastatic PCa tissues and bone metastatic PCa tissues to metastatic bone tissues, and high expression of MAZ was positively correlated with advanced clinicopathological characteristics and poor overall and bone metastasis-free survival in PCa patients. Recurrent gains have been reported to be responsible for MAZ overexpression in a small portion of PCa patients. Furthermore, the upregulation of MAZ promotes while silencing it inhibits invasion and migration of PCa cells in vitro, as well as the bone metastasis ability in vivo. Our results further demonstrated that MAZ activated the RalGEF signalling pathway via transcriptionally activating KRas signaling, which further promoted the bone metastasis of PCa. The clinical correlation revealed that MAZ positively correlated with KRas and RalGEFs signalling activity in PCa and metastatic bone tissues. Collectively, our results unveil a new mechanism responsible for the constitutive activation of the Ras pathway in bone metastasis of PCa, supporting the significance of the transcriptional event in bone metastasis of PCa.

## Methods

### Cell culture

The human PCa cell lines 22RV1, LNCaP, DU145, PC-3, VCaP, and normal.

prostate epithelial cells RWPE-1 were purchased from the Shanghai Chinese Academy of Sciences Cell Bank (China). RWPE-1 cells were cultured in Keratinocyte-SFM (1×) (Invitrogen, USA). PC-3, 22Rv1, and LNCaP cells were grown in RPMI-1640 medium (Life Technologies, USA) supplemented with penicillin G (100 U ml^− 1^), streptomycin (100 mg ml^− 1^) and 10% fetal bovine serum (FBS, Life Technologies, USA). The C4-2B cell line was purchased from the MD Anderson Cancer Center and cultured in T-medium (Invitrogen) supplemented with 10% FBS. DU145 and VCaP cells were grown in Dulbecco’s Modified Eagle’s Medium (Invitrogen, USA) supplemented with 10% FBS [7].

### Plasmid, small interfering RNA and transfection

Human MAZ cDNA was PCR-amplified and cloned into the pMSCV-puro-retro vector (Clontech). Two shRNAs against MAZ,KRas and HRas in the pSuper-puro vector were obtained from Sigma-Aldrich. Transfection of plasmids was performed using Lipofectamine 3000 reagent (Invitrogen, Carlsbad, California, USA) based on the manufacturer’s instructions. Cells (2 × 10^5^) were cultured and infected using a retrovirus produced by pMSCV-puro-MAZ, pSuper-puro-MAZ-shRNA pSuper-puro-KRas-shRNA or pSuper-puro-HRas-shRNA for 3 days. Stable cell lines expressing MAZ, MAZ-shRNAs, MAZ with HRas-shRNAs or MAZ with KRas-shRNAs were selected with 0.5 μg/mL puromycin for 7 days. The human KRas and HRas gene cDNA were obtained from Vigene Biosciences (Shandong, China) and cloned into the pSin-EF2 plasmid (Cambridge, MA, USA). Small interfering RNA (siRNA) for MAZ (siRNA1#: CCUCAACAGUCACGUCAGATT; si RNA2#: AGGUUUUAACGAUUUGUUUTT); KRas (siRNA1#: GCCUUGACGAUACAGCUAATT; siRNA2#: CUAUGGUCCUAGUAGGAAATT) and HRas (siRNA1#: ACACCAAGUCUUUUGAGGATT; siRNA2#: AUGGGAUCACAGUAAAUUATT) consisting of the knockdown and respective scramble RNAs were synthesized and purified by RiboBio. Transfection of plasmids was performed using Lipofectamine 3000 reagent (Invitrogen, Carlsbad, California, USA) in accordance with the manufacturer’s protocol.

### Patients and tumor tissues

The 104 archived fresh specimens, including 53 non-bone metastatic PCa tissues and 36 bone metastatic PCa tissues, and 15 metastatic bone tissues were acquired from The First People’s Hospital of Guangzhou City (Guangzhou, China) between January 2008 and March 2018.To use those clinical specimens for research, patients’ consent and approval from the Institutional Research Ethics Committee were acquired, and in accordance with the Declaration of Helsinki. The 267 paraffin-embedded PCa tissues, including 158 PCa tissues with bone metastasis, 85 PCa tissues without bone metastasis and 24 metastatic bone tissues were also obtained from the First People’s Hospital of Guangzhou City (Guangzhou, China) between 2008 and 2018. The clinicopathological features of the patients are listed in Table 1. The median MAZ expression in PCa tissues was used to distribute the samples by high and low expression of MAZ.

**Table 1:** The relationship between MAZ and clinicopathological characteristics in 243 patients with prostate cancer

Parameters	Number of cases	MAZ expression		*P* values
		Low	High	
Age (years)				
≤ 72	121	68	53	0.124
> 72	122	56	66	
Differentiation				
Well/moderate	96	51	45	0.293
Poor	147	67	80	
Serum PSA				
< 84.7	121	72	49	
> 84.7	122	34	88	< 0.001*
Gleason grade				
≤ 7	102	79	23	
> 7	141	35	106	< 0.001*
Operation				
TURP	71	41	30	
Needle biopsy	204	113	91	
PP	83	39	44	
TURP+BO	35	16	19	
BO	21	9	12	0.419
BM-status				
nBM	158	104	54	
BM	85	18	67	< 0.001*

Abbreviation: *PSA*, Prostate-specific antigen; *TURP*, Trans Urethral Resection Prostate;*PP*, Prior Prostatectomy; *BO*, Bilateral Orchiectomies; *SD*, Standard deviation; *IHC*, Immunological Histological Chemistry; *BM*, Bone Metastasis

### Western blotting

Western blotting was carried out in accordance with a standard method, as previously described. Antibodies against H-Ras (clone-A485), K-Ras (clone-3B10-2F2), N-Ras (clone-2A3), p-ERK1/2(clone-AW39R), ERK (clone-NP2), p-AKT(S473)- polyclonal, p-AKT(T308)-(clone-NL50), AKT (clone-SKB1) and alpha-tubulin (clone-B-5-1-2) were purchased from Sigma-Aldrich (USA).

### Luciferase assay

Cells (4 × 10^4^) were seeded in 24-well plates and cultured for 24 h. Then, Cells were transfected with 250 ng pNFκB-luc, p (CAGAC)12-luc, TOP-Flash, pRaf-luc, EGFR-shc-luc, pGA981–6-luc luciferase reporter plasmid or control plasmids reporter luciferase plasmid, in addition to 5 ng pRL-TK Renilla plasmid (Promega) using Lipofectamine 3000 (Invitrogen) following the manufacturer’s instructions. Both Luciferase and Renilla signals were calculated 36 h after transfection employing a Dual-Luciferase Reporter Assay Kit (Promega) through the manufacturer’s recommendations.

### Transwell assays

The invasion and migration experiments were conducted following a standard method, as described previously. After culturing for 24–48 h, the cells penetrated the coated membrane to the lower surface, where they were fixed with 4% paraformaldehyde and stained with haematoxylin. The cell number was counted under a microscope (× 100).

### RNA extraction, reverse transcription, and real-time PCR

Total RNA from cells or tissues was extracted using Trizol reagent (Invitrogen) following the manufacturer’s protocol. The extracted RNA.

was pretreated with RNase-free DNase, and 2 g of RNA from each sample was used for cDNA synthesis primed with random primer. Complementary DNA (cDNA) was amplified and quantified on a CFX96 real-time system (BIO-RAD, USA) with iQ SYBR Green (BIO-RAD, USA). The primers are listed in Additional file 6: Table. Primers for MAZ, KRas and HRas were purchased from by RiboBio (Guangzhou, China). Glyceraldehyde-3-phosphate dehydrogenase (GAPDH) was conducted as a control. The relative fold expression was calculated using the comparative threshold cycle (2^-ΔΔCt^) method.

### TaqMan copy number assay

A TaqMan CNV assay for MAZ (Hs01274263_cn, Applied Biosystems [ABI]) was conducted through the manufacturer’s instructions. TaqMan CNV reactions were carried out in triplicate with the FAM-dye-labelled assay for MAZ and VIC-dye-labelled RNaseP assay as a reference gene. The relative quantity analysis to calculate copy number was conducted by CopyCaller Software V2.0 (ABI). To confirm the results, all samples were run twice through an independent experiment.

### Animal study

All animal experiments were authorized by The Institutional Animal Care and Use Committee of Sun Yat-sen University. The approval number is L102012017080Q. The laboratory animal welfare was based on the state Standard animal-guideline of the People’s Republic of China. For the animal model, after the anesthetic, BALB/c-nu mice (5–6 weeks old, 18–20 g) were inoculated with 1 × 10^5^ PC-3 cells in 100 μl of PBS through left cardiac ventricle injection. Bone metastases were detected through bioluminescent imaging (BLI) following the previous description [9]. Bone metastasis lesions were indicated on radiographs in the bone as previously described [7]. For survival detection, mice were monitored every day for any indications of discomfort and were either euthanized all at one time or individually when they presented signs of distress, such as 10% loss of body weight, head tilting or paralysis. For the intra-tibia injection animal experiment, BALB/c-nu mice (5–6 weeks old, 18–20 g) were anesthetized using isoflurane, and 2 × 10^6^ VCaP cells in 20 μL were injected into the proximal end of the tibia through a 25-G needle attached to a 100-μL Hamilton syringe [30]. Tumor lesions were monitored by BLI and X-ray.

### Immunohistochemistry

The MAZ, HRas, and KRas expression levels were detected by immunohistochemistry following the previous description [7]. The rating criteria of the specimen are as follows: 0, full negative; 1, < 10% positive; 2, 10–35% positive; 3, 35–75% positive; and 4, > 75% positive. Staining intensity was graded as follows: 0, full negative; 1, weak staining (light yellow); 2, moderate staining (yellow-brown); 3, strong staining (brown). The staining index (SI) was equal to the product of the staining intensity score and the proportion of positive cells. The results of the staining were assessed and scored by two independent pathologists, based on both the proportion of positively stained tumor cell and the intensity of staining. Interobserver reliability was used to examine the consistency of two independent analyses by SPSS statistics 24 software. High and low expression of MAZ were distinguished as follows: SI ≤ 4 defined as low expression of MAZ and SI > 6, defined as high expression of MAZ.

### Chromatin immunoprecipitation (ChIP)

Cells (1 × 10^7^) were cultured in a 10-cm dish,and 1% formaldehyde solution in order was added to cross-link proteins to DNA. Then, the cells were harvested in 1 ml of SDS lysis buffer supplemented with 5 μl of Protease Inhibitor mixture. Cell lysates were Sonicated cells to shear DNA to fragments of 200 to 1000 bp. An equal aliquot of chromatin supernatants was incubated with anti-MAZ antibody (Sigma, #HPA048315), or an anti-IgG antibody (Millipore, Billerica, MA, USA) overnight at 4 °C with rotation. Immunoprecipitation of the DNA–protein complexes were conducted with 60 μl of Protein G Agarose for 1 h at 4 °C. After reversing the cross-linking of protein–DNA complexes to liberate the DNA, the human *KRas or HRas* promoter was amplified by real-time PCR. The primer sequences are listed in Additional file 6: Table S1.

### Affinity pull-down assays for Ral

Cells were cultured in 10-cm tissue dishes and lysed at 75–80% confluency; for tissues, 50–100 mg of fresh tissues were cut into small pieces. Ral activation assays were conducted following the manufacturer’s (Thermo, USA, #88314) protocol.

### Statistical analysis

All values are presented as the mean ± standard deviation (SD). All analyses were performed using GraphPad 7.0 or SPSS 24.0. Student’s t-test was used to determine significant differences between two groups. The chi-square test was used to analyse the relationship between MAZ expression and clinicopathological characteristics. *P* < 0.05 was considered significant. All experiments were repeated three times.

## Results

### MAZ is upregulated in PCa tissues with bone metastasis and further enhanced in metastatic bone tissues

**Fig. 1:**
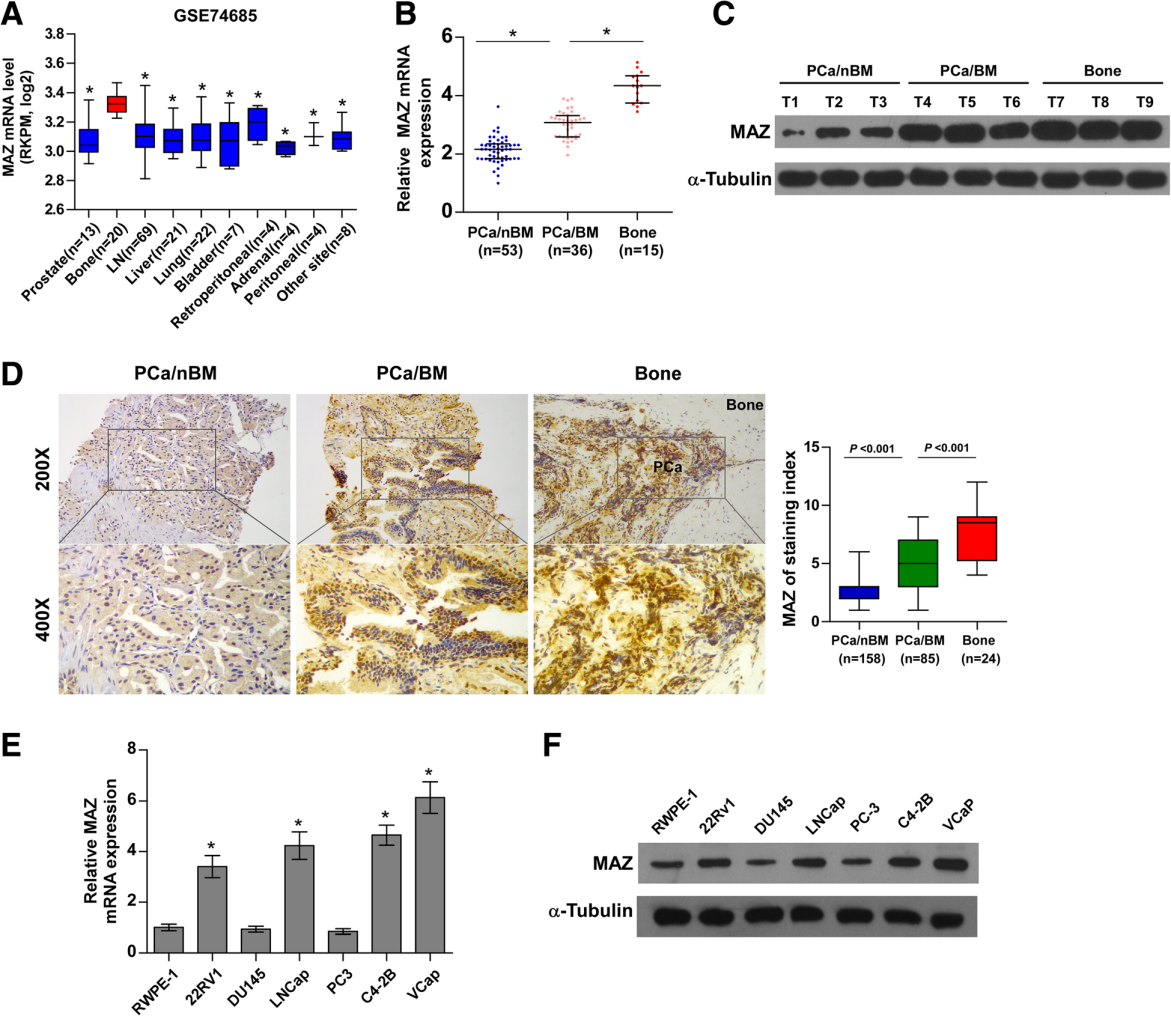
MAZ is upregulated in PCa tissues with bone metastasis and further elevated in metastatic bone tissues. **a** MAZ expression level in metastatic bone tissues derived from PCa was robustly elevated compared with that in primary prostate and other common metastatic sites such as liver, lung, through analyzing the publicly available mRNA sequencing dataset of PCa from GSE74685. *P < 0.05. **b** Real-time PCR analysis of MAZ expression in 89 fresh PCa tissues, including PCa tissues with bone metastases (PCa/BM) and PCa tissues without bone metastases (PCa/nBM), and 15 fresh metastasis bone tissues of PCa (Bone). The case of the lowest normalized CT value of MAZ mRNA was used as a reference whose value defined as 1. The value of all other cases was a multiple of this minimum case. Transcript levels were normalized to GAPDH expression. Lines represent the median and lower/upper quartiles. *P < 0.05. **c** Western blotting analysis of MAZ expression in 3 PCa/nBM, 3 PCa/nBM and 3 bone tissues respectively. We sequentially numbered each group of cases, and then randomly selected 3 cases in each group. α-tubulin served as the loading control. **d** Immunohistochemical (IHC) staining of MAZ protein expression in representative samples of PCa/nBM, PCa/BM and bone were shown. **e** The real-time PCR analysis of MAZ expression levels in the normal prostate epithelial cell (RWPE-1), the non-metastatic PCa cell line 22RV1, bone metastatic PCa cell lines (PC-3, C4-2B, and VCaP) and brain metastatic cell line DU145 and lymph node metastatic cell line LNCaP. Error bars represent the mean ±sd of three independent experiments. *P < 0.05. (F) Western blotting analysis of MAZ expression in PCa cells

As previously reported, the expression level of MAZ in normal bone was relatively lower than that in several other tissues under physiological conditions [25]. Strikingly, we found that MAZ expression was significantly upregulated in metastatic bone tissues derived from PCa compared with primary prostate and other common metastatic sites, such as liver and lung, by analysing the publicly available RNA sequencing dataset of PCa from GSE74685 [31](Fig. 1a). This dramatic differential expression of MAZ between physiological and cancerous situations stimulated our interests to speculate that MAZ may correlate with bone metastasis of PCa. To confirm this hypothesis, 89 fresh PCa tissues, including 53 PCa tissues without bone metastasis (PCa/nBM) and 36 PCa tissues with bone metastasis (PCa/BM), as well as 15 metastatic bone tissues were collected. We further inspected the expression of MAZ in these tissues and found that it was upregulated in PCa/BM compared with PCa/nBM and was further increased in metastatic bone tissues (Fig. 1b). Consistently, Western blot analysis revealed a similar protein expression pattern to the mRNA expression pattern of PCa/nBM, PCa/BM and metastatic bone tissues (Fig. 1c). Immunohistochemical (IHC) staining was performed to validate the expression levels in a large number of PCa and bone samples. As shown in Fig. 1d, MAZ expression was detected in the cell nucleus, and its staining intensity was clearly upregulated in PCa/BM and further increased in metastatic bone tissues. The mRNA and protein levels of MAZ in PCa cell lines were further detected. Compared with the normal epithelial prostate cell line RWPE-1, we found that MAZ expression was significantly upregulated in the non-metastatic PCa cell line 22RV1 from a xenograft of CWR22R cells, the lymph node metastatic PCa cell line LNCaP and the bone metastatic PCa cell line VCaP and C4-2B but not in the brain metastatic PCa cell line DU145 and the bone metastatic PCa cell line PC-3 (Fig. 1e and f). These results implicate that the high expression of MAZ is correlated with bone metastasis of PCa.

### Recurrent gains are the potential mechanism responsible for MAZ overexpression in a part of PCa tissues

**Fig. 2:**
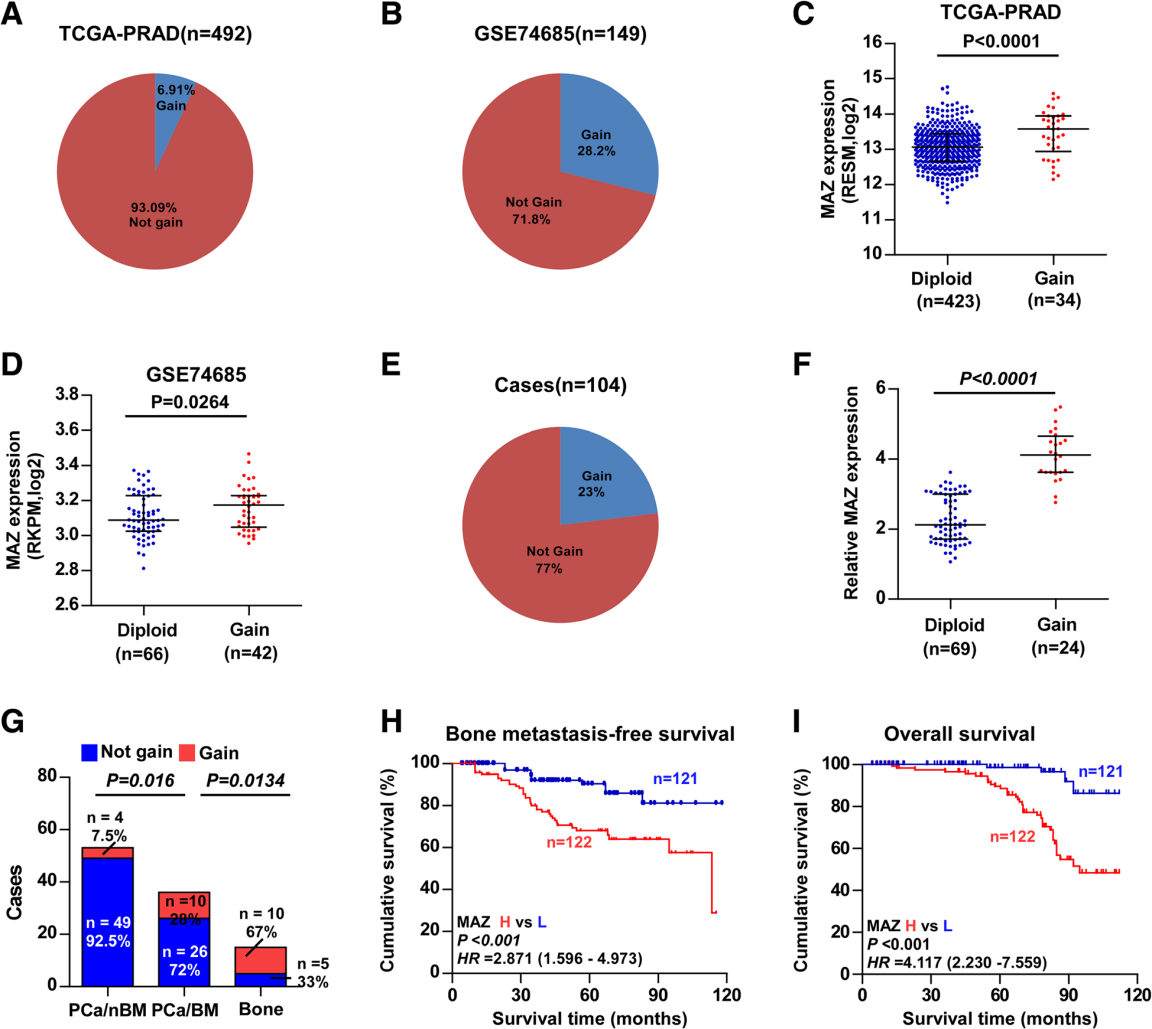
Recurrent gains are the underlying mechanism responsible for MAZ overexpression. **a, b** The percentage of MAZ with gains in the PCa samples from TCGA-PRAD and GSE74685. **c, d** The average expression level of MAZ in PCa tissues with gains exceeded those with diploid in TCGA-PRAD and GSE74685 samples. Each bar stands for median values ± quartile values. *P < 0.05. **e** The percentage of MAZ with gains in our PCa samples. **f** The average expression level of MAZ in PCa samples with gains and metastasis bone tissues of PCa samples with gains exceeded those samples with diploid gains. Each bar stands for median values ± quartile values. **g** Percentages and numbers of MAZ samples with gains in PCa samples with distinct bone metastasis status and in metastasis bone samples of PCa. **h** Kaplan-Meyer analysis of our PCa patients bone metastasis-free survival between the MAZ-overexpression (*n* = 122) and MAZ-downexpression (*n* = 121) group (Log-rank Test). **i** Kaplan-Meyer analysis of our PCa patients overall survival between the MAZ-overexpression (*n* = 122) and MAZ-downexpression (*n* = 121) group (Log-rank Test)

To investigate the mechanism responsible for MAZ overexpression in PCa tissues, we further analysed the PCa dataset from TCGA-PRAD [32] and GSE74685 and found that the frequencies of recurrent gains (amplification) in TCGA-PRAD and GSE74685 were 6.91 and 28.2% respectively (Fig. 2a and b). In both datasets, the expression level of MAZ in cases with gains was robustly elevated compared with those with the diploid (Fig. 2c and d). Furthermore, we measured the gains levels in our own PCa cases using a TaqMan copy number assay [33] and found that gains were found in 24/104 PCa cases (approximately 23%) (Fig. 2e), and the expression level of MAZ was considerably higher in cases with gains than in those with diploid (Fig. 2f). Importantly, gains occurred in 10/15 PCa metastatic bone tissues (approximately 67%) and in 10/36 PCa tissues with bone metastasis (approximately 28%), but only in 4 out of 53 PCa tissues without bone metastasis (approximately 7.5%) (Fig. 2g). These results show that recurrent gains are responsible for MAZ overexpression in PCa tissues.

### High expression of MAZ correlates with poor bone metastasis-free survival in PCa patients

To understand in-depth the clinical significance of MAZ in PCa patients, the clinical correlation between MAZ expression and clinicopathological characteristics in PCa patients was first analysed. As shown in Table 1, MAZ overexpression positively correlated with serum PSA levels, Gleason grade and bone metastasis status in PCa patients. Kaplan-Meier survival analysis showed that PCa patients with high MAZ expression presented a shorter overall survival (OS) compared with those with low MAZ expression, as well as poor bone metastasis-free survival (Fig. 2h and i). Furthermore, high expression of MAZ predicted a poor OS and progression-free survival in PCa patients based on analysis of the PCa dataset from TCGA-PRAD (Additional file 1: Figure S1). These findings reveal that overexpression of MAZ correlates with poor prognosis as well as bone metastasis and progression status in PCa patients.

### MAZ promotes bone metastasis of PCa cells

**Fig. 3:**
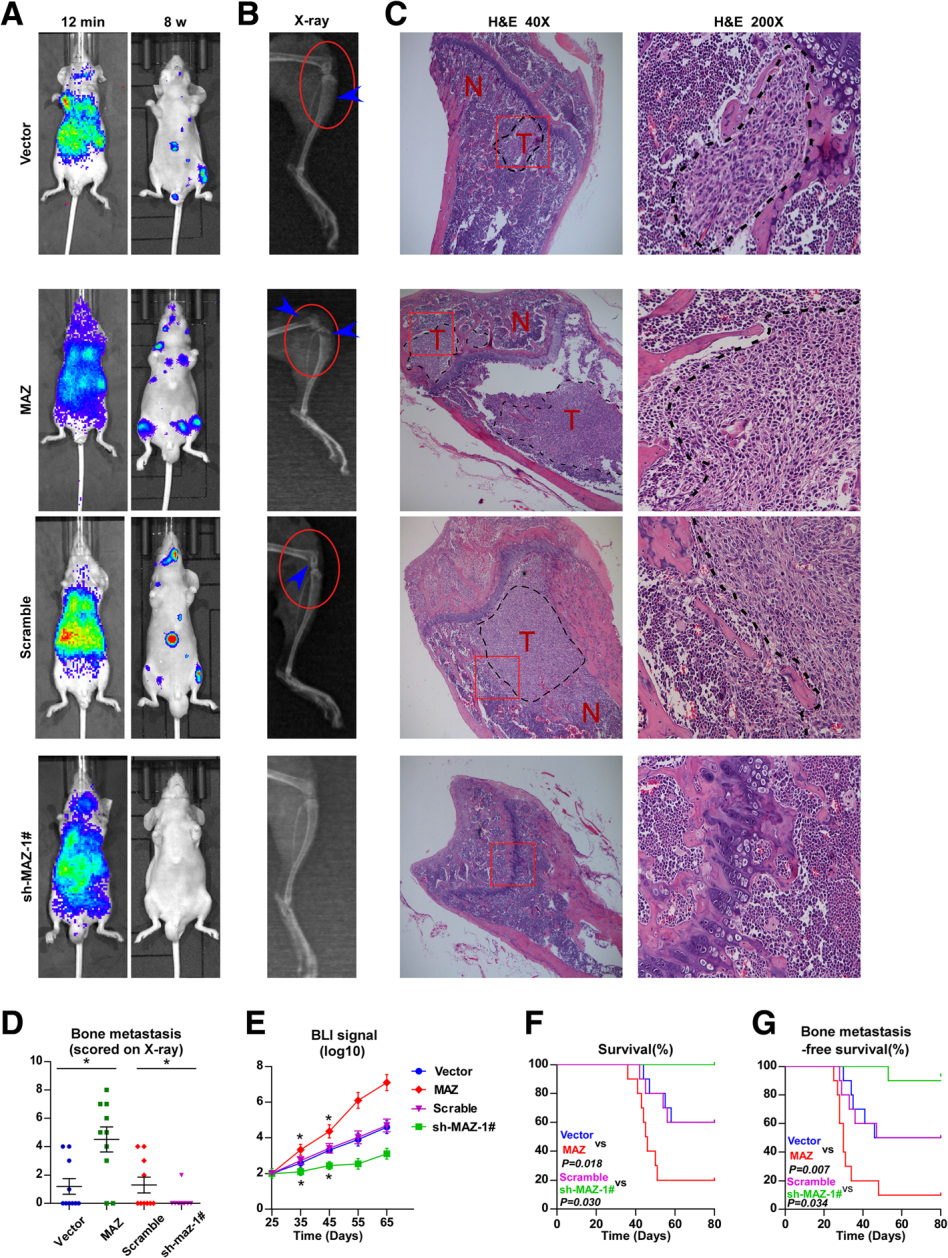
MAZ promotes bone metastasis of PC-3 cells **a** Representative BLIs signal of bone metastasis of a mouse from the indicated groups of mice at 12 mins and 8 weeks respectively. **b** Representative radiographic images of bone metastases in the indicated mice (arrows indicate osteolytic lesions). **c** Representative H&E-stained sections of tibias from the indicated mouse. **d** The sum of bone metastasis score for each mouse in tumor-bearing mice inoculated with vector (*n* = 10), MAZ (n = 10), scramble(n = 10) and sh-MAZ-1# (n = 10) cells. *P < 0.05. **e** Quantification of the BLI signaling in the vector, MAZ, scramble and sh-MAZ-1# groups at 25, 35,45,55 and 65 days respectively. *P < 0.05. T-test was not conducted on days 55 and 65 due to the death of a large number of mice. **f** Kaplan-Meyer analysis of mouse survival in the vector, MAZ, scramble and sh-MAZ-1# groups (Gehan-Breslow-Wilcoxon Test). **(G)** Kaplan-Meyer analysis of mouse bone metastasis-free survival in the vector, MAZ, scramble and sh-MAZ-1# groups (Gehan-Breslow-Wilcoxon Test)

To investigate the role of MAZ in the bone metastasis of PCa, we first constructed MAZ stably expressing cell lines by ectopically overexpressing MAZ and endogenously silencing MAZ in PC-3 by retrovirus infection. (Additional file 2: Figure S2A, C). We also generated MAZ-silencing constructs in VCaP by sh-MAZ plasmid transfection. (Additional file 2: Figure S2B, C). Invasion and migration assays showed that upregulation of MAZ escalated, while silencing MAZ reduced the invasion and migration ability of PCa cells (Additional file 2: Figure S2D-G). However, overexpression of MAZ in VCaP cells has no significant effect on their invasion and migration abilities (data not shown). A mouse model of bone metastasis was used (*n* = 10 mice per group), in which the luciferase-labelled vector, MAZ-overexpressing PC-3 cells, scramble, and MAZ-silencing PC-3 cells were inoculated into the left cardiac ventricle of male nude mice to monitor the development of distant bone metastasis loci by BLI and X-ray. As shown in Fig. 3a and b, the MAZ-overexpressing PC-3 cells group suffered more bone metastases compared with the vector group, while the MAZ-silencing PC-3 cells group displayed fewer bone metastases compared with the scramble group by BLI and X-ray. H&E staining of tumor sections from the tibias of inoculated mice showed that upregulation of MAZ significantly aggravated while downregulation of MAZ clearly reduced the tumor burden in bone (Fig. 3c). Furthermore, MAZ-overexpressing PC-3 cells exhibited more metastatic foci and severe osteolytic areas of metastatic tumors, as well as a shorter survival and bone metastasis-free survival compared with the vector group; conversely, the mouse group injected with the MAZ-silenced PC-3 cells yielded the opposite results (Fig.3d-g). To explore the effect of MAZ on AR-positive PCa cells in vivo, an intra-tibial injection model (n = 10 mice per group) was used to observe the effect of silencing MAZ on the tumorigenic ability of VCaP cells. As shown in Additional file 2: Figure S2 H-I, the mice injected with MAZ-silenced VCaP cells displayed less bone tumor lesion compared with the control group by BLI and X-ray. H&E staining of the tumors section from the tibias of inoculated mice showed that downregulating MAZ obviously remitted the tumor burden in bone (Additional file 2: Figure S2 J). At the same time, MAZ-silencing VCaP cells exhibited less osteoblastic area of bone tumors, as well as a longer bone tumor burden-free survival time compared with the scramble group (Additional file 2: Figure S2 K-L). These results demonstrate that MAZ promotes bone metastasis of PCa cells in vivo and enhances invasion and migration abilities of PCa cells in vitro.

### MAZ transcriptionally activates RAS signalling

**Fig. 4:**
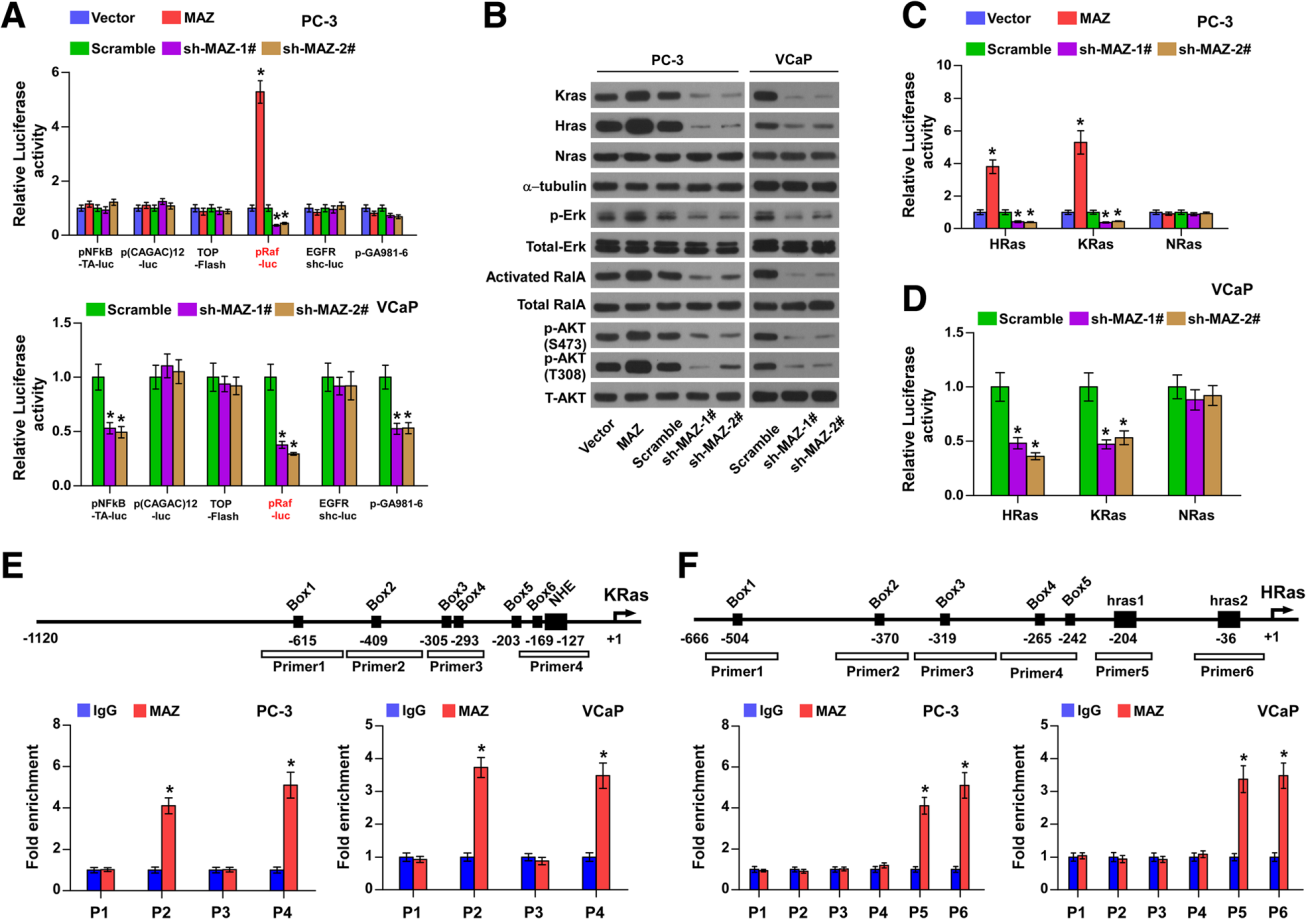
MAZ transcriptionally activates RAS signaling. **a** The luciferase reporter activity of the NF-kB, TGF-β, EGFR, WNT, Notch and RAS pathway were analyzed in PC3 and VCaP. Luciferase reporter plasmids were employed to detect the activity of the NF-kB, TGF-β, EGFR, WNT, Notch and RAS pathways in PC-3 transfected with overexpression of MAZ or sh-MAZ plasmid, in VCaP transfected with si-MAZ plasmid. *P < 0.05. **b** Western blot analysis of HRas, KRas, NRas, p-ERK, activated RalA, p-AKT(S473), p-AKT(T308) in MAZ-overexpressing and MAZ-silencing cells. The total ERK, total RalA, and α-tubulin were used as a loading control. **c,d** The luciferase activity was monitored by transfection full-length HRas, KRas, or NRas promoter in vector, MAZ-overexpression, scramble, or MAZ-silencing PCa cells. Error bars represent such mean ± S.D. of three independent experiments. *P < 0.05. **e, f** Schematic illustration of the promoter regions of human KRas and HRas gene, and the regions containing the primers used for the ChIP assay (upper). ChIP was performed using an anti-MAZ antibody or a control IgG to identify MAZ binding sites in the KRas and HRas promoter. *P < 0.05.

To determine the underlying specific signalling involved in the pro-bone metastasis role of MAZ in PCa, we further examined the effects of MAZ on multiple well-documented bone metastasis-related signalling pathways, including the TGF-β [7], Wnt [8], NF-kB [9], EGFR [10], and Notch [11] signalling, using a luciferase reporter assay. As shown in Fig. 4a, the luciferase reporter activity of Ras signalling was upregulated by overexpression of MAZ in PC-3 cells and consistently repressed by silencing of MAZ in two bone metastatic PCa cells. The three Ras genes, Hras, Kras and Nras, have been reported to be the most common oncogenes in human cancer [34, 35]. Thus, we further investigated the effects of MAZ on these three Ras protein family members in PCa cells by qPCR and Western blot analysis. We found that MAZ upregulation dramatically increased while silencing remarkably inhibited the KRas and HRas expression in PCa cells, but had no effect on NRas expression in PCa cells (Fig. 4b and Additional file 3: Figure S3A-B). Furthermore, we explored the expression of MAZ, KRAS, and HRAS in each group of bone metastasis animal model in Fig. 3. The analysis of IHC staining showed that upregulating MAZ enhanced, while silencing MAZ repressed HRAS and KRAS expression in the bone tumor tissues from tibia in different mice groups, confirming the positive correlation of MAZ with HRAS and KRAS in these PCa cells (Additional file 3: Figure S3C-F). Three critical downstream effector signalling pathways of Ras activity, namely-ERK, PI3K/AKT, and RalGEFs, were equally examined. Phospho-ERK1/2 and phosphor-AKT were considered as indicators of ERK and PI3K/AKT signalling activation in PCa cells, respectively. The RalGEF function was measured by determining the level of its enzymatic product, RalA-GTP, using a pull-down assay [36]. As shown in Fig. 4b, overexpression of MAZ raised the levels of p-Erk, p-AKT at S473 and T308 and activated RalA in PCa cells, whereas MAZ silencing had the opposite effect.

To unveil the specific mechanism by which MAZ activates RAS signalling in PCa cells, we generated three luciferase reporter constructs containing an approximately 1- kb region of the KRas and HRas promoters, respectively. As shown in Fig. 4c-d, KRas and HRas promoter activity were enhanced by co-transfection with the MAZ-overexpressing plasmid in PC-3 cells and decreased in MAZ-silenced PCa cells compared with the respective empty vector-transfected control cells. Additionally, we performed chromatin immunoprecipitation (ChIP) assays to identify the regions of the KRas and HRas promoter that might be bound by MAZ. Previous studies have indicated that the promoter region of the KRas contains a GC box and nuclease-hypersensitive element (NHE) region [37], and the promoter region of the HRAS also contains a GC box and two G-rich elements (hras-1 and hras-2) [38, 39], all of which are easily bound by the transcription factor. Therefore, according to the characteristics of its promoter region, 4 and 6 pairs of primers were designed for the promoter regions of KRas and HRas, respectively. The results showed that region 2 and 4 of the KRas promoter had the highest affinity for MAZ protein (Fig. 4e), while regions 5 and 6 of the HRas promoter had the highest affinity (Fig. 4f), implying that MAZ upregulates KRas and HRas at the transcriptional level. Taken together, our results indicate that MAZ transcriptionally activates RAS signalling in PCa cells.

### KRas is essential for the pro-bone metastasis role of MAZ in vivo

**Fig. 5:**
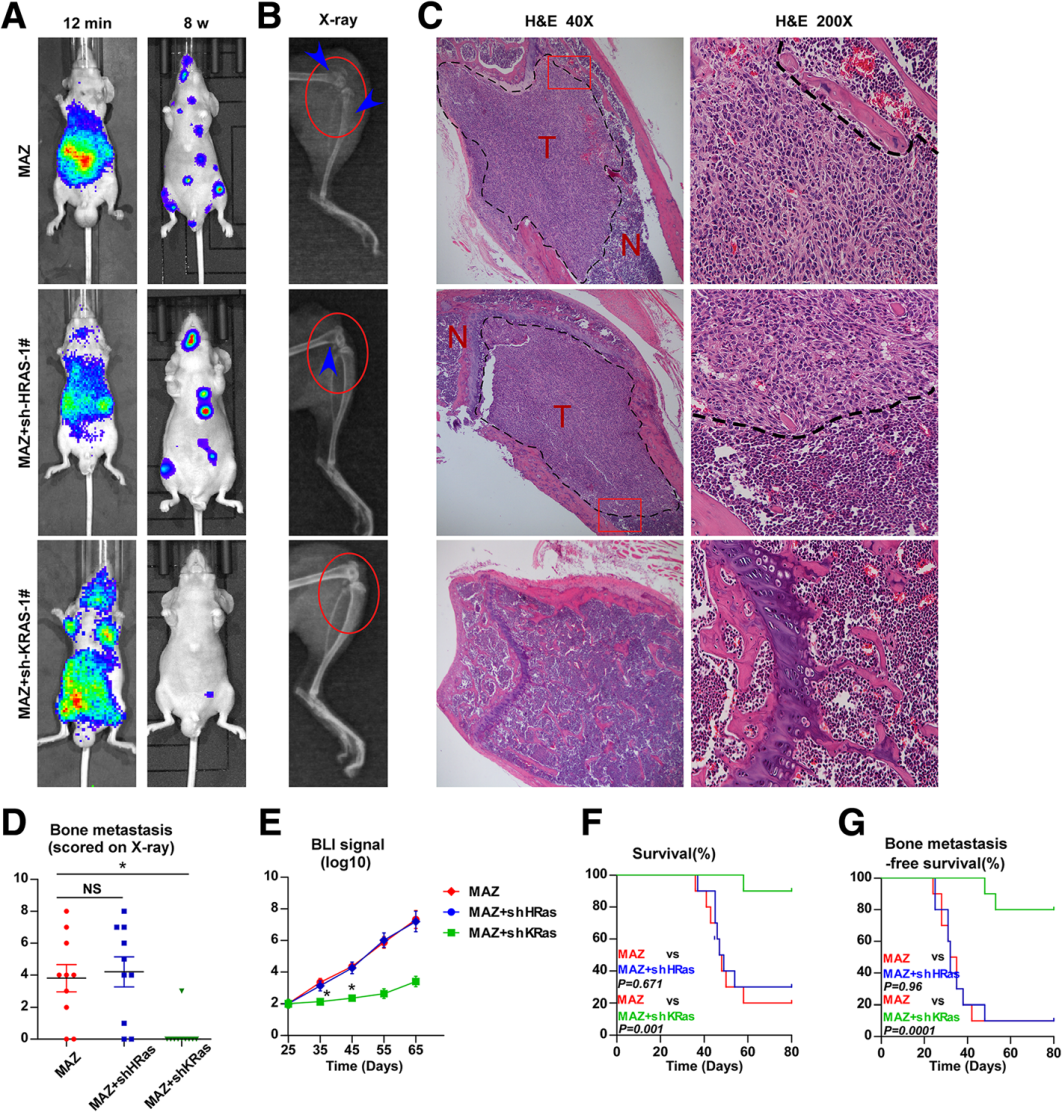
KRas is essential for the pro-bone metastasis role of MAZ in vivo. **a** Representative BLIs signal of bone metastasis of a mouse from the indicated groups of mice at 12 mins and 8 weeks respectively. **b** Representative radiographic images of bone metastases in the indicated mice (arrows indicate osteolytic lesions). **c** Representative H&E-stained sections of tibias from the indicated mouse. **d** The sum of bone metastasis score for each mouse in tumor-bearing mice inoculated with MAZ-overexpression (*n* = 10), MAZ-overexpression and KRas-silencing (*n* = 10), and MAZ-overexpression and HRas-silencing (*n* = 10) cells. *P < 0.05. **e** Quantification of the BLI signaling in MAZ-overexpression, MAZ-overexpression, and KRas-silencing, and MAZ-overexpression and HRas-silencing groups at 25, 35, 45, 55 and 65 days respectively. *P < 0.05. T-test was not performed on days 55 and 65 owing to the death of a large number of mice. **f** Kaplan-Meyer analysis of mouse survival in the MAZ-overexpression, MAZ-overexpression, and KRas-silencing, and MAZ-overexpression and HRas-silencing groups (Gehan-Breslow-Wilcoxon Test).**(G)** Kaplan-Meyer analysis of mouse bone metastasis-free survival in the MAZ-overexpression, MAZ-overexpression, and KRas-silencing, and MAZ-overexpression and HRas-silencing groups (Gehan-Breslow-Wilcoxon Test)

As demonstrated above, MAZ transcriptionally activates KRas and HRas in PCa cells. Next, we considered whether one or both mediated the pro-metastatic role of MAZ overexpression in vitro and in vivo. A gene set enrichment analysis (GSEA) of MAZ based on mRNA expression data from the TCGA-PRAD revealed that the MAZ expression level was strongly and positively correlated with the KRas pathway (Additional file 3: Figure S3G, H). And then we constructed MAZ-overexpression with sh-HRas and MAZ-overexpression with sh-KRas stably expressing cell lines by endogenously silencing HRas or KRas in MAZ-overexpression PC-3 by retrovirus infection (Additional file 4: Figure S4A-D). The mice were divided into three groups: MAZ-overexpressing group, MAZ-overexpressing with KRas-silencing group and MAZ-overexpressing with HRas-silencing group. As shown in Fig. 5a-g, we found that KRas silencing markedly abrogated the pro-bone metastasis ability in MAZ-overexpressing PC-3 cells, including a decrease in the bone metastatic score and osteolytic area of tumors and a longer bone metastasis-free survival and OS compared with the MAZ-overexpressing group by BLI, X-ray, H&E staining and survival analysis. However, silencing of HRas had no significant effect on the bone metastasis ability of MAZ-overexpressing PC-3 cells in vivo (Fig. 5a-g). Consistently, invasion and migration assays showed that silencing KRas rescued the invasion and migration abilities of MAZ-overexpressing PC-3 cells (Additional file 4: Figure S4E, F). Thus, these results indicate that MAZ promotes bone metastasis of PCa by transcriptionally activating KRas.

### MAZ promotes bone metastasis of PCa cells in a manner dependent on KRas-mediated RalGEFs signalling

**Fig. 6:**
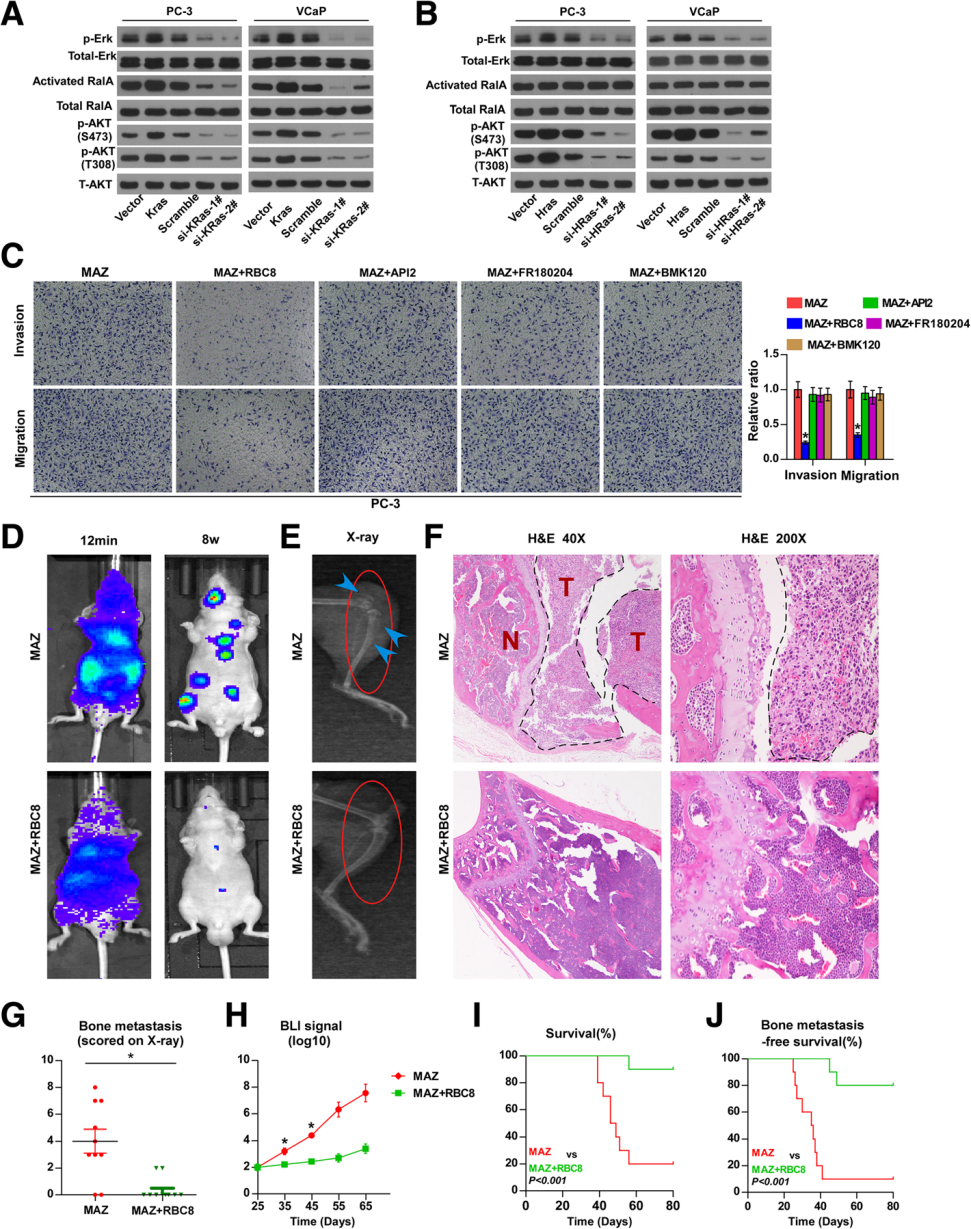
MAZ promotes bone metastasis of PCa cells dependent on KRas-mediated RalGEFs signaling **a, b** Western blot analysis of p-ERK, activated RalA, p-AKT(S473), p-AKT(T308) in the indicated cell lines. The total ERK, total RalA, and α-tubulin were used as a loading control. **c** The RalGEFs pathway inhibitor RBC8 reduced migration and invasive abilities of MAZ-overexpression PC-3 cells, but the AKT, PI3k and ERK pathway inhibitors API2, BMK120 and FR180204 did not. The dose and duration of use of these three inhibitors were 3 μM (RBC8) for 48 h, 1 uM (API2) for 24 h, 1 uM (BMK120) for 24 h and 50 uM (FR180204) for 72 h, respectively. Error bars represent the mean ± S.D. of three independent experiments. *P < 0.05.**d** Representative BLIs signal of bone metastasis of a mouse from the indicated groups of mice at 12 mins and 8 weeks respectively. RBC8 was dissolved in DMSO and injected i.p. daily at 50 mg/kg/day which initiated 24 h after inoculation of PC-3 cells in the left cardiac ventricle. Mice in the control group were injected with the appropriate amount of solvent per day. **e** Representative radiographic images of bone metastases in the indicated mice (arrows indicate osteolytic lesions). **f** Representative H&E-stained sections of tibias from the indicated mouse. **g** The sum of bone metastasis score for each mouse in tumor-bearing mice inoculated with MAZ-overexpression (*n* = 10) and MAZ-overexpression with RBC8 (*n* = 10) cells. *P < 0.05. **h** Quantification of the BLI signaling in the MAZ-overexpression and MAZ-overexpression with RBC8 groups at 25, 35, 45, 55 and 65 day respectively. *P < 0.05. T-test was not performed on days 55 and 65 owing to the death of a large number of mice. **i** Kaplan-Meyer analysis of mouse survival in the MAZ-overexpression and MAZ-overexpression with RBC8 groups (Gehan-Breslow-Wilcoxon Test). **j** Kaplan-Meyer analysis of mouse bone metastasis-free survival in the MAZ-overexpression and MAZ-overexpression with RBC8 groups (Gehan-Breslow-Wilcoxon Test)

Previous studies have demonstrated that different Ras isoforms exhibited quantitative and qualitative differences in their ability to activate particular downstream effector signalling [15, 40]. To discern the underlying signalling responsible for the different roles of KRas and HRas in mediating the pro-bone metastasis of MAZ overexpression in PCa, the effects of KRas and HRas on the expression levels of p-Erk, p-AKT and activated RalA were further investigated by constructing KRas or HRas expressing PCa cell lines by ectopically overexpressing KRas or HRas in PC-3 cells and endogenously knocking down KRas or HRas by retrovirus infection in PC-3 and VCaP cell lines (Additional file 5: Figure S5A-C). Western blot analysis revealed that the upregulation or silencing of KRas simultaneously increased or reduced the expression levels of p-Erk, p-AKT and activated RalA in PCa cells (Fig. 6a). However, the upregulation or silencing of HRas enhanced or inhibited the expression levels of p-Erk and p-AKT but had no influence on activated RalA (Fig. 6b). Moreover, KRas, but not HRas, had a significant effect on the invasion and migration abilities of PCa cells. (Additional file 5: Figure S5A-G). Our aforementioned results demonstrated that MAZ transcriptionally activated KRas and HRas, where only silencing KRas can rescue the pro-bone metastasis effects of MAZ overexpression in PCa (Figs. 4 and 5). Thus, these findings suggest that RalGEFs signalling may be a potential effector signalling contributing to the different roles of KRas and HRas in mediating the pro-bone metastasis of MAZ overexpression in PCa. To verify this hypothesis, three inhibitors of the activity of the three major effector pathways downstream of Ras (ERK, AKT, and RalGEFs pathway), including RBC8 for RalGEFs [41], FR180204 for ERK [42], BMK120 [43] for PI3K and API2 for AKT [44], were applied to MAZ-overexpressing PC-3 cells. Predictably, only RBC8 effectively attenuated the stimulatory effects of MAZ overexpression on the invasion and migration ability of PC-3 cells (Fig. 6c). Importantly, RBC8 dramatically repressed the bone metastasis ability in MAZ-overexpressing cells (Fig. 6d-j). Collectively, our results indicate that MAZ promotes bone metastasis of PCa via KRas/RalGEFs signalling.

### Clinical correlation of MAZ with KRas and activated RalA in human PCa tissues

**Fig. 7:**
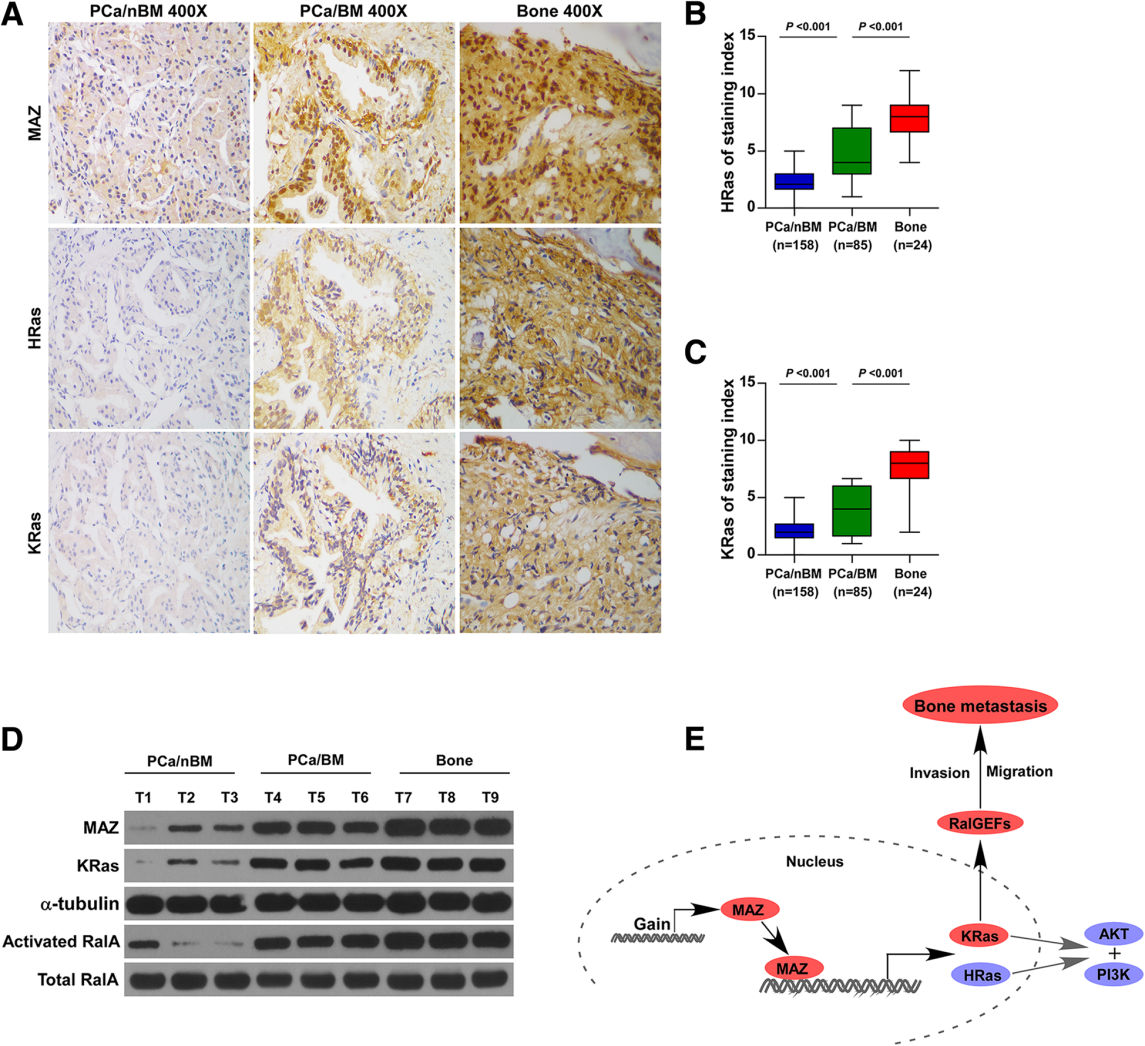
Clinical correlations of MAZ with KRas and activated RalA in human PCa tissues. **a-c** Immunohistochemical (IHC) staining of MAZ, KRas and HRas protein expression in representative samples of PCa/nBM, PCa/BM and bone were shown. **d** Analysis of MAZ expression with KRas and activated RalA in 3 bone metastatic PCa tissues, 3 nonbone metastatic PCa tissues, and 3 metastatic bone tissues. We sequentially numbered each group of cases, and then randomly selected 3 cases in each group. Total RalA and α-tubulin were used as a loading control. **e** Hypothetical model illustrating that overexpression of MAZ activates KRas and RalGEFS, leading to the bone metastasis of PCa

To investigate the clinical significance of MAZ-induced KRas and HRas, IHC staining was performed in the PCa tissues. As shown in Fig. 7a-c, MAZ expression in PCa tissues with bone metastasis was elevated compared with that in PCa tissues without bone metastasis and further increased in metastatic bone tissues; consistently, the expression of HRas and KRas exhibited the same pattern. Since activated RAL cannot be observed by IHC, it is not presented here. Meanwhile, the protein expression levels of MAZ, KRas and activated RalA were examined in PCa tissues and metastatic bone tissues by western blotting. As shown in Fig. 7d, MAZ expression was elevated in PCa tissues with bone metastasis (T4–6) compared with expression in PCa tissues without bone metastasis and was further increased in metastatic bone tissues (T7–9); consistently, protein expression of KRas and activated RalA exhibited the same pattern (Fig. 7d). Taken together, our results indicate that overexpression of MAZ activates KRas and RalGEFS, resulting in the bone metastasis of PCa (Fig. 7e).

## Discussion

As a transcription factor, the upregulation of MAZ has been extensively implicated in tumorigenesis, progression, and metastasis in various types of cancer [26, 27, 29]. In PCa, a study from Jiao et al. has shown that overexpression of MAZ contributes to proliferation and metastasis of PCa through reciprocal regulation of androgen receptor [26], suggesting that MAZ may play a role in the metastatic phenotype of PCa. However, the clinical significance and biological role of MAZ in bone metastasis of PCa have not been reported until now. In this study, we found that MAZ was elevated in bone metastatic PCa tissues compared with nonbone metastatic PCa tissues, and it was further enhanced in metastatic bone tissues, which positively correlated with advanced clinicopathological characteristics and poor overall and bone metastasis-free survival in PCa patients. Interestingly, the expression level of MAZ was relatively lower in normal bone than in several other tissues under physiological conditions [25]. This finding greatly ignites our interest in further exploring the mechanism responsible for the dramatic differential expression of MAZ between physiological and cancerous situations, which was demonstrated to be associated with recurrent gains in the current study. Next, our results revealed that the upregulation of MAZ promoted while its silencing inhibited the invasion and migration of PCa cells in vitro, as well as the bone metastasis ability in vivo. Our results further demonstrate that MAZ promoted bone metastasis of PCa via transcriptionally activating Ras signalling. Thus, these findings indicate that MAZ functions as a pro-bone metastasis gene in PCa. Indeed, several lines of evidence have demonstrated that the metastatic potential of prostate cancer depends on the expression of several metastasis-related genes, or metastasis-promoting genes [45–47], which further determine the pivotal role of MAZ in bone metastasis of PCa.

As shown in the CHIP experiments Fig. 4e, MAZ bind to thenuclease-hypersensitive element (NHE) of KRas’ promoter which activated the KRas transcription. Theoretically, KRas mutation in this site will block the transcriptional activation of KRas by MAZ and thus affect the occurrence of bone metastasis of prostate cancer. Although Ras signalling was constitutively activated by mutations in approximately 30% of human cancers [18], Ras mutations were very rare in PCa [19, 48], suggesting that other regulatory mechanisms are involved in aberrant activation of Ras signalling in PCa. It is worth noting that transcriptional regulation is reported to be another primary mechanism responsible for the activation of Ras signalling in several types of cancer [20–22]. Multiple studies have revealed that MAZ plays an important role in the activation of RAS signalling by varying mechanisms in several tumor types [20, 22, 49]. For example, MAZ promoted angiogenesis through transcriptional activation of the RAS signalling pathway in breast cancer [22]. In addition, HRas is silenced by two neighbouring G-quadruplexes and activated by MAZ in bladder cancer cells [49]. Interestingly, Yu et al reported that MAZ mediated by FOXF2 plays dual roles in basal-like breast cancer: promotion of proliferation and suppression of progression [50]. However, whether these Ras protein members are separately or simultaneously activated by MAZ in bone metastasis of PCa, and whether activation of these Ras signalling members produces subsequent functional roles in mediating the effects of MAZ on bone metastasis of PCa, need to be further elucidated. In the current study, our results demonstrated that MAZ simultaneously transcriptionally activated KRas and HRas signalling in bone metastatic PCa cells. Importantly, our results demonstrated that KRas, but not HRas, was indispensable to the pro-bone metastasis role of MAZ in PCa. Therefore, our results reveal a new mechanism responsible for the sustained activation of Ras signalling in bone metastasis of PCa.

The primary downstream effector pathways of Ras signalling include ERK, PI3K and RalGEFs [15, 18], each of which has been reported to play important roles in bone metastasis of PCa [12, 16, 17]. In this study, we reported that MAZ simultaneously activated ERK, PI3K, and RalGEFs signalling in PCa cells, where ERK and PI3K signalling were under concomitant regulation of KRas and HRas, and RalGEFs were only regulated by KRas. Strikingly, our results showed that KRas silencing markedly abrogated the invasion and migration abilities of MAZ-overexpressing PC-3 cells in vitro and pro-bone metastasis ability in vivo; however, silencing HRas had no significant effect on the bone metastasis ability of MAZ-overexpressing PC-3 cells. These findings support the notion that RalGEFs signalling may be a potential downstream effector signalling contributing to the pro-bone metastasis of MAZ in PCa. In fact, RalGEFs inhibitor RBC8 dramatically suppressed the invasion and migration abilities of MAZ-overexpressing PC-3 cells. However, ERK inhibitor or PI3K inhibitors had no obvious influence on the invasion and migration abilities in MAZ-overexpressing PC-3 cells. More importantly, RBC8 dramatically inhibited the bone metastasis ability of MAZ-overexpressing PCa cells. Therefore, our results reveal that MAZ promotes bone metastasis of PCa cells in a manner that is dependent on KRas-mediated RalGEFs signalling, although ERK and PI3K/AKT signalling are hyperactive in MAZ-overexpressing PCa cells.

## Conclusion

Our results unveil that overexpression of MAZ elicited by recurrent gains activates the Kras/ RalGEFs signalling pathway, which further promotes bone metastasis in PCa, providing theoretical evidence that the MAZ/Kras/ RalGEFs signalling axis plays an important role in bone metastasis of PCa. Therefore, a comprehensive understanding of the mechanism responsible for the activation of the Ras signalling pathway will facilitate the development of an effective therapeutic strategy to inhibit bone metastasis of PCa.

## Additional files

Additional file 1: **Figure S1.** High expression of MAZ correlates with poor overall survival and progression-free survival in PCa patients (A) Kaplan-Meyer analysis of patients overall survival in TCGA-PRAD between the MAZ-overexpression (*n* = 247) and MAZ-downexpression (n = 247) group. (B) Kaplan-Meyer analysis of patients progression-free survival between the MAZ-overexpression (*n* = 209) and MAZ-downexpression (*n* = 217) group. (TIF 180 kb)

Additional file 2: **Figure S2.** Overexpressing enhanced, while silencing MAZ repressed invasion and migration in PCa cells. (A) The real-time PCR analysis of MAZ expression in PC-3 cells transduced with MAZ or sh-MAZ plasmid compared to vector or scramble. (B) The real-time PCR analysis of MAZ expression in VCaP cells transduced with sh-MAZ plasmid compared to vector. Transcript levels were normalized by GAPDH expression. Error bars represent the mean ± s.d. of three independent experiments. *P < 0.05. (C) Western blotting analysis of MAZ expression in MAZ-overexpressing or MAZ-silencing PCa cells. (D, E) Overexpression of MAZ enhanced, while silencing MAZ suppressed invasion and migration abilities in PC-3 cells. Error bars represent the mean ± S.D. of three independent experiments. *P < 0.05. (F, G) Silencing MAZ suppressed invasion and migration abilities in VCaP cells. Error bars represent the mean ± S.D. of three independent experiments. *P < 0.05. (H) Representative BLIs signal of tibia tumor lesion of a mouse from the indicated groups of mice at 12 mins and 6 weeks respectively. (I) Representative radiographic images of bone tumor lesion in the indicated mice (arrows indicate lesions). (J) Representative H&E-stained sections of tibias from the indicated mouse. (K) The sum of bone tumor score for each mouse in tumor-bearing mice inoculated with scramble(*n* = 10) and sh-MAZ-1# (n = 10) VCaP cells. *P < 0.05 **(L)** Kaplan-Meyer analysis of mouse bone tumor burden-free survival in the scramble and sh-MAZ-1# groups (Gehan-Breslow-Wilcoxon Test). (TIF 7437 kb)

Additional file 3: **Figure S3.** MAZ expression significantly and positively correlated with the KRas signaling. (A-B) The real-time PCR analysis of HRas, KRas and NRas mRNA expression levels in MAZ-overexpressing and MAZ-silencing cells**.** Error bars represent the mean s.d. of three independent experiments. *P < 0.05. (C-F) Immunohistochemical (IHC) staining of MAZ, KRas and HRas protein expression in representative samples of tumor-bearing mice inoculated with vector (n = 10), MAZ (n = 10),scramble(n = 10) and sh-MAZ-1# (n = 10) cells. (G, H) Gene set enrichment analysis (GSEA) revealed that MAZ expression significantly and positively correlated with the KRas signaling. (TIF 29292 kb)

Additional file 4 **Figure S4.** Silencing KRas rescued the invasion and migration abilities of MAZ-overexpressing PC-3 cells. (A, B) The real-time PCR analysis of KRas or HRas expression in MAZ-overexpression PC-3 cells transduced with sh-KRas or sh-HRas plasmid compared to scramble. Transcript levels were normalized by GAPDH expression. Error bars represent the mean ± s.d. of three independent experiments. *P < 0.05. (C, D) Western blotting analysis of KRas or HRas expression in the indicated cells. α-Tubulin was used as the loading control. (E) Silencing KRas rescued the invasion and migration abilities of MAZ-overexpressing PC-3 cells, while silencing HRas did not. Error bars represent the mean ± s.d. of three independent experiments. *P < 0.05. (TIF 2311 kb)

Additional file 5: **Figure S5**. KRas had a significant effect on the invasion and migration abilities of PCa cells. (A) The real-time PCR analysis of HRas expression in PC-3 and VCaP cells transduced with overexpression-HRas or si-HRas plasmid compared to vector or scramble. Transcript levels were normalized by GAPDH expression. Error bars represent the mean ± s.d. of three independent experiments. *P < 0.05. (B) The real-time PCR analysis of KRas expression in PC-3 and VCaP cells transduced with overexpression-KRas or si-KRas plasmid compared to vector or scramble. Transcript levels were normalized by GAPDH expression. Error bars represent the mean ± s.d. of three independent experiments. *P < 0.05. (C) Western blotting analysis of KRas or HRas expression in indicated cells. (D, E) Overexpression of KRas enhanced, while silencing KRas suppressed invasion and migration abilities in PC-3 and VCaP cells. Error bars stand for mean ± S.D. of three independent experiments. *P < 0.05. (F, G) No matter overexpressed HRas or silenced HRas had no effect on migration and invasion of PC-3 and VCaP. Error bars represent the mean ± S.D. of three independent experiments. *P < 0.05. (TIF 487 kb)

Additional file 6: **Table S1** A list of primers used in the KRas CHIP assay. **Table S2**.A list of primers used in the HRas CHIP assay. **Table S3**. A list of primers used in the reactions for real-time RT-PCR (DOCX 16 kb)

## Data Availability

The data about MAZ analyzed in this article are available among TCGA-PRAD and GSE74685 (TCGA website: http://www.cbioportal.org/study?id=prad_tcga; GSE74685: http://www.cbioportal.org/study?id=prad_fhcrc#summary). The Gene Set Enrichment Analysis (GSEA) was run by GSEA 2.2.1 (http://www.broadinstitute.org/gsea) and gene set was performed by Molecular Signatures Database v5.2 (http://software.broadinstitute.org/gsea/msigdb).

## Abbreviations

BLI: bioluminescence imaging
ChIP: Chromatin immunoprecipitation
EGFR: Epidermal growth factor receptor;
ERK: Extracellular signal-regulated kinase
GAPDH: Glyceraldehyde-3-phosphate dehydrogenase
GSEA: Gene set enrichment analysis
H&E: Hematoxylin and Eosin Stain
MAZ: Myc-associated zinc-finger protein
MEK: Extracellular-signal regulated kinase kinase
NF-kB: Nuclear factor kappa-light-chain-enhancer of activated B cells
PI3K: Phosphatidylinositol 3-kinase
RalGEF: Ral Guanine nucleotide exchange factor
TCGA-PRAD: The Cancer Genome Atlas-Prostate adenocarcinoma
TGF-β: Transforming growth factor-β
