# Supplementary figures and images for: MAZ promotes prostate cancer bone metastasis through transcriptionally activating the KRas-dependent RalGEFs pathway
Source: J Exp Clin Cancer Res. 2019 Sep 5;38:391. doi: 10.1186/s13046-019-1374-x (PMC6729064; doi:10.1186/s13046-019-1374-x)

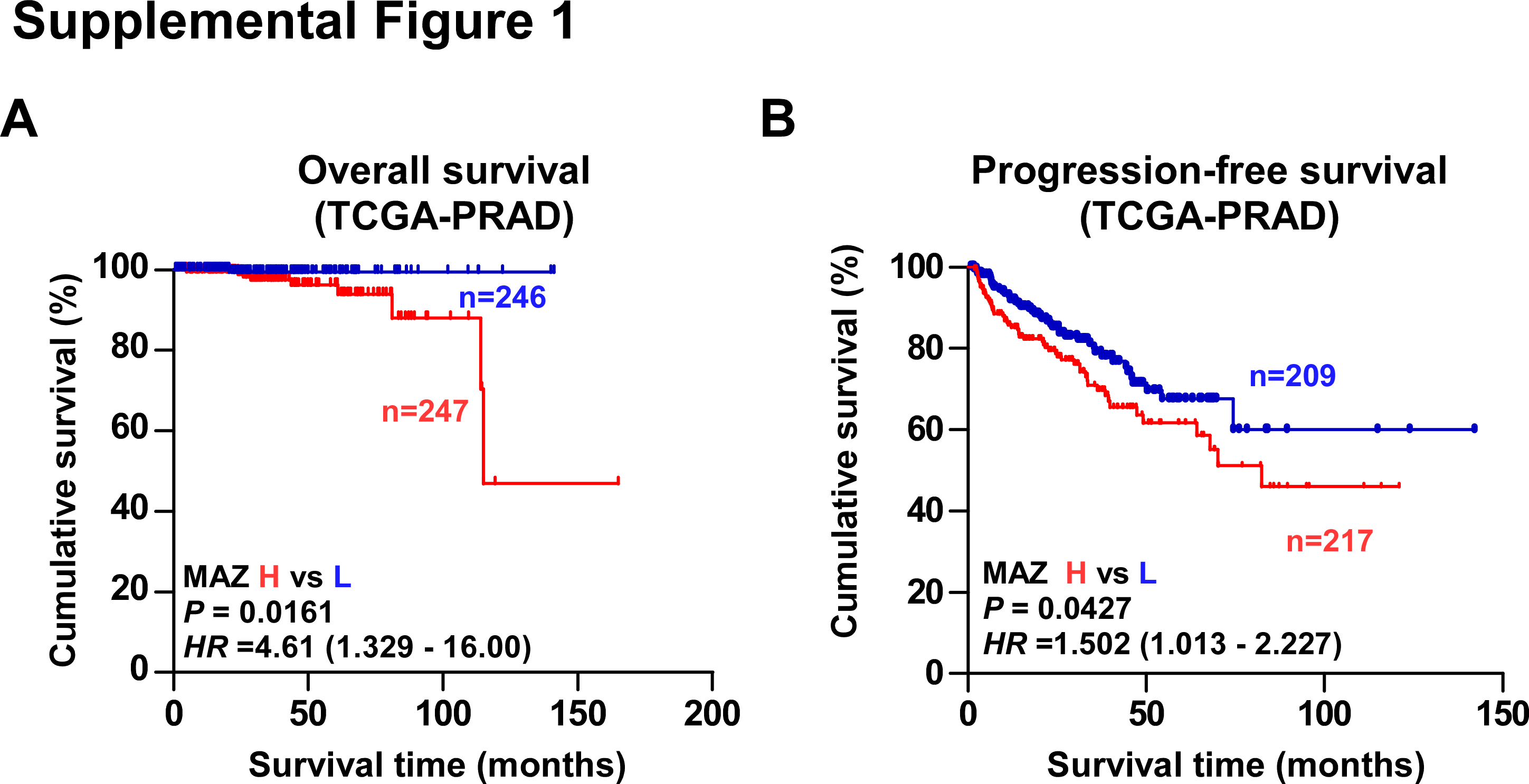

Supplement: Supplementary file 1 — Figure S1. High expression of MAZ correlates with poor overall survival and progression-free survival in PCa patients (A) Kaplan-Meyer analysis of patients overall survival in TCGA-PRAD between the MAZ-overexpression (n = 247) and MAZ-downexpression (n = 247) group. (B) Kaplan-Meyer analysis of patients progression-free survival between the MAZ-overexpression (n = 209) and MAZ-downexpression (n = 217) group. (TIF 180 kb) [file 13046_2019_1374_MOESM1_ESM.tif]

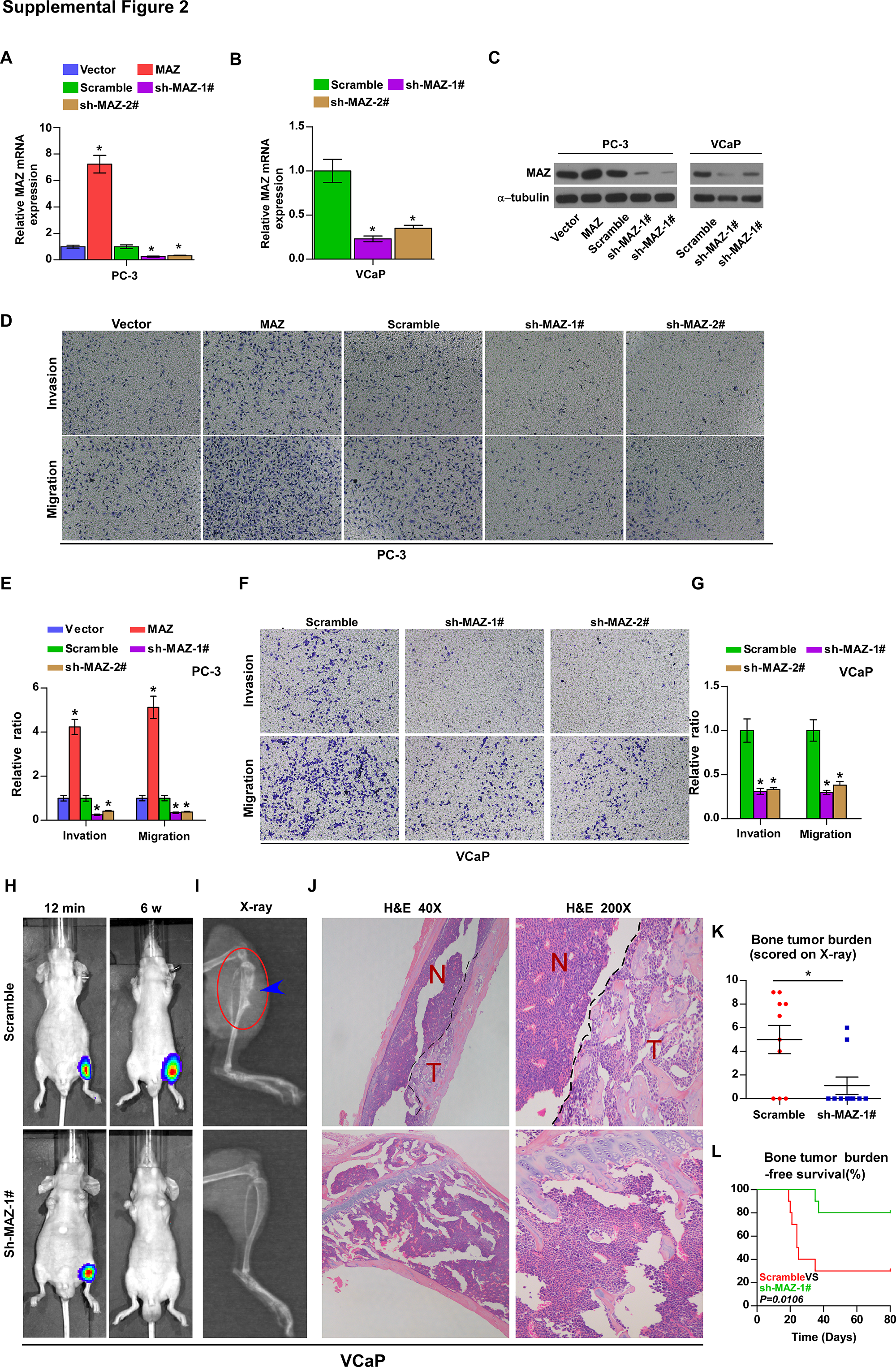

Supplement: Supplementary file 2 — Figure S2. Overexpressing enhanced, while silencing MAZ repressed invasion and migration in PCa cells. (A) The real-time PCR analysis of MAZ expression in PC-3 cells transduced with MAZ or sh-MAZ plasmid compared to vector or scramble. (B) The real-time PCR analysis of MAZ expression in VCaP cells transduced with sh-MAZ plasmid compared to vector. Transcript levels were normalized by GAPDH expression. Error bars represent the mean ± s.d. of three independent experiments. *P < 0.05. (C) Western blotting analysis of MAZ expression in MAZ-overexpressing or MAZ-silencing PCa cells. (D, E) Overexpression of MAZ enhanced, while silencing MAZ suppressed invasion and migration abilities in PC-3 cells. Error bars represent the mean ± S.D. of three independent experiments. *P < 0.05. (F, G) Silencing MAZ suppressed invasion and migration abilities in VCaP cells. Error bars represent the mean ± S.D. of three independent experiments. *P < 0.05. (H) Representative BLIs signal of tibia tumor lesion of a mouse from the indicated groups of mice at 12 mins and 6 weeks respectively. (I) Representative radiographic images of bone tumor lesion in the indicated mice (arrows indicate lesions). (J) Representative H&E-stained sections of tibias from the indicated mouse. (K) The sum of bone tumor score for each mouse in tumor-bearing mice inoculated with scramble(n = 10) and sh-MAZ-1# (n = 10) VCaP cells. *P < 0.05 (L) Kaplan-Meyer analysis of mouse bone tumor burden-free survival in the scramble and sh-MAZ-1# groups (Gehan-Breslow-Wilcoxon Test). (TIF 7437 kb) [file 13046_2019_1374_MOESM2_ESM.tif]

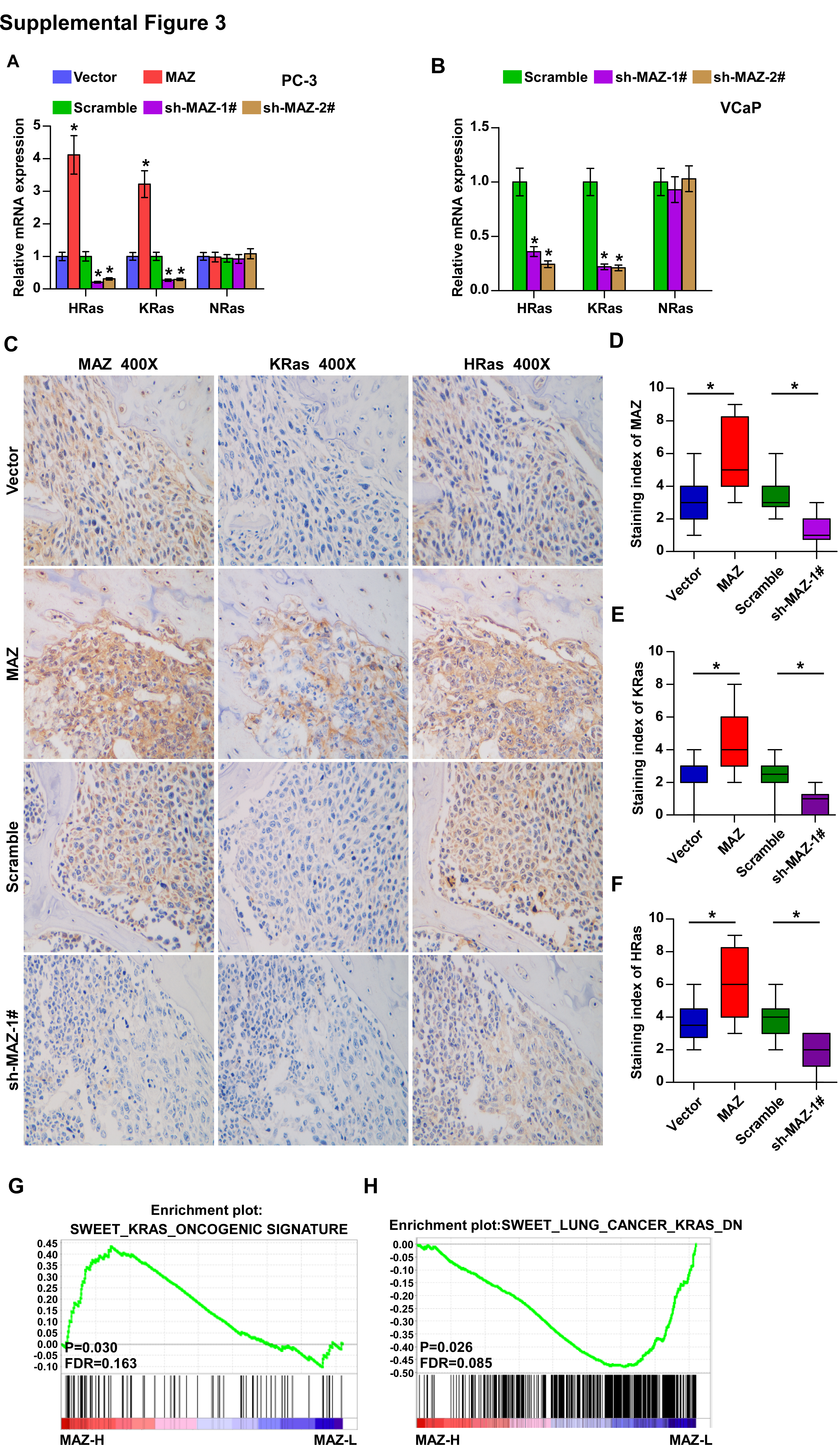

Supplement: Supplementary file 3 — Figure S3. MAZ expression significantly and positively correlated with the KRas signaling. (A-B) The real-time PCR analysis of HRas, KRas and NRas mRNA expression levels in MAZ-overexpressing and MAZ-silencing cells. Error bars represent the mean s.d. of three independent experiments. *P < 0.05. (C-F) Immunohistochemical (IHC) staining of MAZ, KRas and HRas protein expression in representative samples of tumor-bearing mice inoculated with vector (n = 10), MAZ (n = 10),scramble(n = 10) and sh-MAZ-1# (n = 10) cells. (G, H) Gene set enrichment analysis (GSEA) revealed that MAZ expression significantly and positively correlated with the KRas signaling. (TIF 29292 kb) [file 13046_2019_1374_MOESM3_ESM.tif]

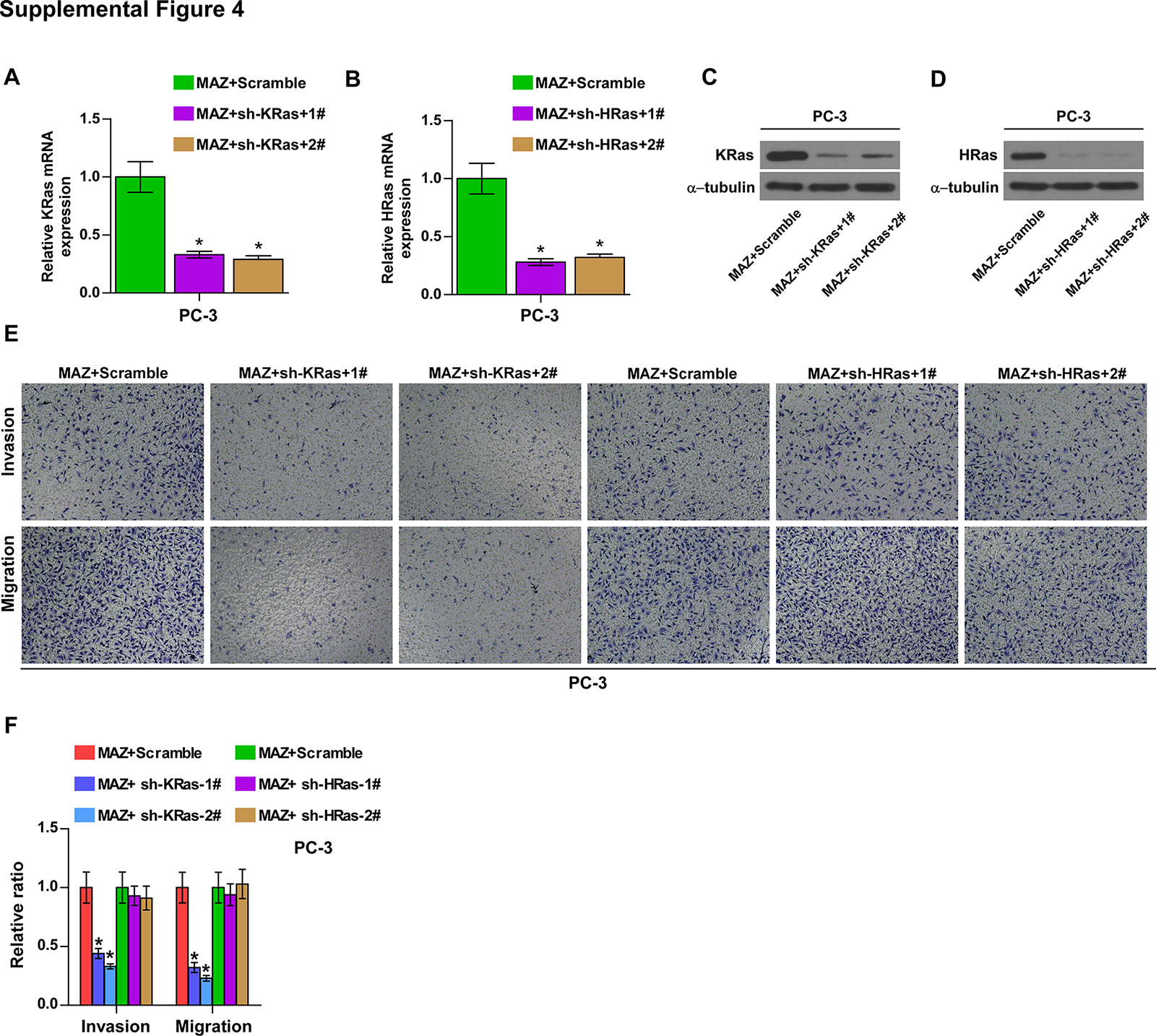

Supplement: Supplementary file 4 — Figure S4. Silencing KRas rescued the invasion and migration abilities of MAZ-overexpressing PC-3 cells. (A, B) The real-time PCR analysis of KRas or HRas expression in MAZ-overexpression PC-3 cells transduced with sh-KRas or sh-HRas plasmid compared to scramble. Transcript levels were normalized by GAPDH expression. Error bars represent the mean ± s.d. of three independent experiments. *P < 0.05. (C, D) Western blotting analysis of KRas or HRas expression in the indicated cells. α-Tubulin was used as the loading control. (E) Silencing KRas rescued the invasion and migration abilities of MAZ-overexpressing PC-3 cells, while silencing HRas did not. Error bars represent the mean ± s.d. of three independent experiments. *P < 0.05. (TIF 2311 kb) [file 13046_2019_1374_MOESM4_ESM.tif]

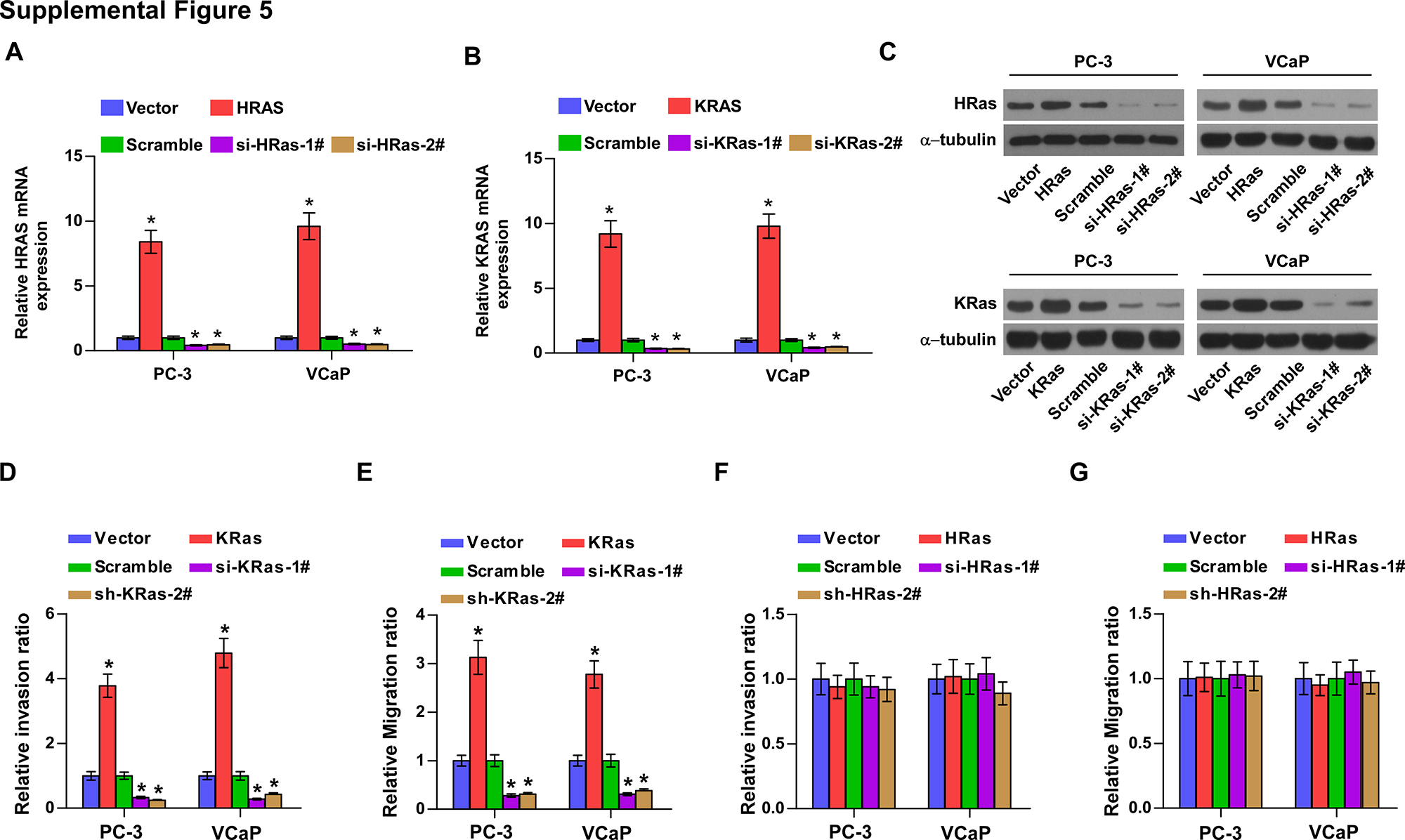

Supplement: Supplementary file 5 — Figure S5. KRas had a significant effect on the invasion and migration abilities of PCa cells. (A) The real-time PCR analysis of HRas expression in PC-3 and VCaP cells transduced with overexpression-HRas or si-HRas plasmid compared to vector or scramble. Transcript levels were normalized by GAPDH expression. Error bars represent the mean ± s.d. of three independent experiments. *P < 0.05. (B) The real-time PCR analysis of KRas expression in PC-3 and VCaP cells transduced with overexpression-KRas or si-KRas plasmid compared to vector or scramble. Transcript levels were normalized by GAPDH expression. Error bars represent the mean ± s.d. of three independent experiments. *P < 0.05. (C) Western blotting analysis of KRas or HRas expression in indicated cells. (D, E) Overexpression of KRas enhanced, while silencing KRas suppressed invasion and migration abilities in PC-3 and VCaP cells. Error bars stand for mean ± S.D. of three independent experiments. *P < 0.05. (F, G) No matter overexpressed HRas or silenced HRas had no effect on migration and invasion of PC-3 and VCaP. Error bars represent the mean ± S.D. of three independent experiments. *P < 0.05. (TIF 487 kb) [file 13046_2019_1374_MOESM5_ESM.tif]
