# Supplementary material for: MAZ promotes prostate cancer bone metastasis through transcriptionally activating the KRas-dependent RalGEFs pathway
Source: J Exp Clin Cancer Res. 2019 Sep 5;38:391. doi: 10.1186/s13046-019-1374-x (PMC6729064; doi:10.1186/s13046-019-1374-x)
Supplement: Supplementary file 6 — Table S1 A list of primers used in the KRas CHIP assay. Table S2.A list of primers used in the HRas CHIP assay. Table S3. A list of primers used in the reactions for real-time RT-PCR (DOCX 16 kb) [file 13046_2019_1374_MOESM6_ESM.docx]

Supplemental Table 1. A list of primers used in the KRas CHIP assay

| KRas CHIP assay primers | |
| --- | --- |
| Primer1 - Forward | AGCAGTCACCAAAAGTGGGA |
| Primer1- Reverse | TGATAATAATCGGGGCGGCG |
| Primer2 - Forward | GCAGCCGCCAATTCTGACC |
| Primer2- Reverse | CTTCGCAGCTTCTCTGTGGAG |
| Primer3 - Forward | CGGTCTAGGGTGGCGAG |
| Primer3- Reverse | GGACAGCCTTGCGGCTA |
| Primer4 - Forward | CAGACGGGCGTACGAGA |
| Primer4- Reverse | GTCCGGCAGTCCCTCCT |

Supplemental Table 2.A list of primers used in the HRas CHIP assay

| HRas CHIP assay primers | |
| --- | --- |
| Primer1- Forward | GACCGGAAGGAGCTCGTTG |
| Primer1- Reverse | GCCGCTCTCTTGGGCTG |
| Primer2- Forward | GAGTCGGTTGGCCCCAC |
| Primer2- Reverse | GAGAGCCCCAACCCGAAC |
| Primer3- Forward | GCGTCGGTTACCATCCGG |
| Primer3- Reverse | GCAGACAGTCCTCGGCG |
| Primer4- Forward | GTCGGTTACCATCCGGCG |
| Primer4- Reverse | GGCAGACAGTCCTCGGC |
| Primer5- Forward | CCTGCCTCGGGTACGC |
| Primer5- Reverse | CGCCGGATGGTAACCGAC |
| Primer6- Forward | GCGGACACTTGCCACTCAC |
| Primer6- Reverse | CGCCGGATGGTAACCGA |

Supplemental Table 3. A list of primers used in the reactions for real-time RT-PCR

| Real-time PCR primer | |
| --- | --- |
| MAZ- Forward | GGATCACCTCAACAGTCACGTC |
| MAZ- Reverse | GGCACTTTCTCCTCGTGTCGTA |
| GAPDH- Forward | GTCTCCTCTGACTTCAACAGCG |
| GAPDH- Reverse | GGCACTTTCTCCTCGTGTCGT |
| KRAS- Forward | GGATCACCTCAACAGTCACGTC |
| KRAS- Reverse | GGCACTTTCTCCTCGTGTCGTA |
| HRAS-Forward | ACGCACTGTGGAATCTCGGCAG |
| HRAS-Reverse | TCACGCACCAACGTGTAGAAGG |
